# Chromosome evolution in Lophyohylini (Amphibia, Anura, Hylinae)

**DOI:** 10.1371/journal.pone.0234331

**Published:** 2020-06-11

**Authors:** Pablo Suárez, Juan M. Ferro, Cleusa Y. Nagamachi, Dario E. Cardozo, Ailin Blasco-Zúñiga, Jéssica B. Silva, Euvaldo Marciano-JR, Marco A. Costa, Victor G. D. Orrico, Mirco Solé, Igor J. Roberto, Miryan Rivera, John E. Wiley, Julián Faivovich, Diego Baldo, Julio C. Pieczarka

**Affiliations:** 1 Instituto de Biología Subtropical (CONICET-UNaM), Puerto Iguazú, Misiones, Argentina; 2 Laboratorio de Genética Evolutiva "Claudio J. Bidau", Instituto de Biología Subtropical (CONICET-UNaM), Facultad de Ciencias Exactas, Químicas y Naturales, Universidad Nacional de Misiones, Posadas, Misiones, Argentina; 3 Laboratório de Citogenética, Centro de Estudos Avançados da Biodiversidade, Instituto de Ciências Biológicas, Universidade Federal do Pará, Belém, Pará, Brasil; 4 Laboratorio de Investigación de Citogenética y Biomoléculas de Anfibios (LICBA), Centro de Investigación para la Salud en América Latina-CISeAL, Facultad de Ciencias Exactas y Naturales, Pontificia Universidad Católica del Ecuador, Quito, Ecuador; 5 Departamento de Ciências Biológicas, Universidade Estadual de Santa Cruz, Ilhéus, Bahia, Brazil; 6 Centro de Conservação e Manejo de Fauna da Caatinga, Cemafauna-Caatinga, Petrolina, Pernambuco, Brazil; 7 Departamento de Ciências Biológicas, Pós-graduação em Zoologia, Universidade Federal do Amazonas, Amazonas, Brazil; 8 Department of Pediatrics/Medical Genetics, East Carolina University School of Medicine, Greenville, NC, United States of America; 9 División Herpetología, Museo Argentino de Ciencias Naturales “Bernardino Rivadavia”—CONICET, Buenos Aires, Argentina; 10 Departamento de Biodiversidad y Biología Experimental, Facultad de Ciencias Exactas y Naturales, Universidad de Buenos Aires, Buenos Aires, Argentina; Leibniz-Institute of Freshwater Ecology and Inland Fisheries, GERMANY

## Abstract

The hyline tribe Lophyohylini includes 87 species of treefrogs, of which cytogenetics aspects have been studied in less than 20% of them. In order to evaluate the evolution of some of its chromosome characters (NOR position, C-bands, and DAPI/CMA_3_ bands), we studied the karyotypes of 21 lophyohylines, 16 of them for the first time, and analyzed them in a phylogenetic context. Most species showed similar karyotypes regarding chromosome number (2n = 24) and morphology (FN = 48), excepting *Phyllodytes edelmoi* and *Osteocephalus buckleyi* with 2n = 22 (FN = 44) and 2n = 28 (FN = 50), respectively. The NOR location was variable among species and provided valuable phylogenetic information. This marker was located in pair 11 in all species of *Trachycephalus*, *Itapotihyla langsdorffii*, and *Nyctimantis arapapa*, representing the plesiomorphic condition of Lophyohylini. Besides, other apomorphic states were recovered for the clades comprising *N*. *rugiceps* and *N*. *siemersi* (NOR in pair 5), and *Dryaderces pearsoni*, *Osteocephalus*, and *Osteopilus* (NOR in pair 9). *Phyllodytes* presented variation for NORs position; they were in pair 2 in *P*. *edelmoi*, pair 7 in *P*. *melanomystax*, and pair 8 in *P*. *gyrinaethes* and *P*. *praeceptor*. Polymorphisms in size, number, and activity of this marker were observed for *N*. *siemersi*, *Osteocephalus fuscifacies*, and some species of *Trachycephalus*. Remarkably, in *N*. *siemersi* NORs were detected on a single chromosome in the two specimens studied by this technique, raising the question of how this complex polymorphism is maintained. Interstitial telomeric sequences were found in *P*. *edelmoi*, *P*. *melanomystax*, and *Osteocephalus buckleyi*, and their presence seems to be not related to the chromosome reorganization events. Finally, some species showed spontaneous rearrangements, possibly as a consequence of an uncommon phenomenon in anuran cytogenetics: the presence of fragile sites or secondary constrictions not associated with NORs. We propose that this rare feature would have played an important role in the evolution of this group of frogs. From the evidence obtained in this and previous studies, we conclude that Lophyohylini presents a complex chromosome evolution.

## Introduction

Treefrogs of the subfamily Hylinae are essential components of Neotropical anuran diversity, currently comprising 724 species [1], arranged in seven tribes [2]: Cophomantini, Dendropsophini, Hylini, Lophyohylini, Pseudini, Scinaxini, and Sphaenorhynchini. Lophyohylini consists of 87 species that are widely distributed in Middle and South America [1, 3]. The monophyly of this tribe is well supported, mostly by molecular characters [3–11]. Blotto et al. [3] recently performed the most inclusive phylogenetic analysis for Lophyohylini and recovered three major clades: (1) An early diverging one composed of *Itapotihyla* and *Phytotriades*; (2) A clade including *Trachycephalus*, closely related to *Corythomantis* and *Nyctimantis* (redefined by them to include all species formerly in *Aparasphenodon*, *Argenteohyla* and one species of *Corythomantis*), and (3) A clade composed of *Tepuihyla* as a sister taxon of *Dryaderces* and *Osteocephalus*, plus *Osteopilus* and *Phyllodytes*. Although the monophyly of all recognized genera currently in Lophiohylini is well-supported by previous studies, it is not the case for its major clades that are still poorly supported.

Several contributions have studied chromosome evolution in different clades of Hylinae (e.g., [12–17]). More recently, Schmid et al. [18] summarized most cytogenetic information known for Hylidae, further contributing with novel karyotypes for 14 species of Hylinae (2 Cophomantini, 2 Hylini, 2 Scinaxini, and 8 Dendropsophini).

The available chromosomal information for Lophyohylini is very sparse and restricted to only 19% of the included taxa, corresponding to 16 species of 7 genera [19–22]. All studied species have diploid karyotypes, being the most extended haploid number n = 12 [22], proposed as a synapomorphy of Hylinae [4,17].

Among Lophyohylini, the karyotypes of *Osteopilus* and *Phyllodytes* are distinctive regarding the haploid number and morphology of chromosomes. Fusion and fission rearrangements, involving the plesiomorphic karyotype, were proposed as possible mechanisms for explaining the variability described in the tribe [19,22]. In *Os*. *wilderi* (n = 14) and *Os*. *ocellatus* (n = 17), Cole [23] and Anderson [19] reported increments in chromosome number, resulting in the occurrence of two and ten pairs of telocentric chromosomes, respectively. On the other hand, a reduction in chromosome number was observed in *Phyllodytes edelmoi* and *P*. *luteolus* (n = 11, [22]). Interestingly, in the former species, Gruber et al. [22] reported interstitial telomeric sequences or ITS on pairs 1 and 2. Other remarkable features reported in Lophyohylini are the presence of fragile sites or interstitial secondary constrictions that are not associated with NORs in *Osteocephalus* [19] and conspicuous NORs size heteromorphisms in several species of the genera *Corythomantis*, *Itapotihyla*, *Nyctimantis*, *Phyllodytes*, and *Trachycephalus* [21,22].

The main goal of this study was to increase the knowledge about the chromosome evolution of hylids, focusing mainly on Lophyohylini. For this, we surveyed different aspects of the chromosome characters, including the number and morphology of chromosomes, differential banding staining (C-banding, Ag-NORs, DAPI and CMA_3_ fluorochromes), and mapping of both ribosomal and telomeric DNA repetitive sequences by fluorescence *in situ* hybridizations. We analyzed the karyotypes of 21 species of Lophyohylini, 16 for the first time. Results were interpreted and discussed based on the most recent and inclusive phylogenetic hypothesis for this tribe [3].

## Material and methods

We analyzed karyotypes of 50 specimens of 21 species of Lophyohylini belonging to the genera *Dryaderces* (*D*. *pearsoni*), *Itapotihyla* (*I*. *langsdorffii*), *Nyctimantis* (*N*. *arapapa*, *N*. *rugiceps*, and *N*. *siemersi*), *Osteocephalus* (*O*. *buckleyi*, *O*. *fuscifacies*, *O*. *leprieurii*, *O*. *planiceps*, *O*. *oophagus*, and *O*. *taurinus*), *Osteopilus* (*Os*. *septentrionalis* and *Os*. *vastus*), *Phyllodytes* (*P*. *edelmoi*, *P*. *gyrinaethes*, *P*. *praeceptor*, and *P*. *melanomystax*), *Trachycephalus* (*T*. *dibernardoi*, *T*. *helioi*, *T*. *jordani*, and *T*. *typhonius*). The collection, euthanization, and preservation of specimens were carried out with the approval of the Ethical Committee in Animal Use (CEUA–permission numbers 002/12 and 014/15 UESC, Ilhéus, BA, Brazil) and the following institutions: Argentina, Administración de Parques Nacionales (APN, PD-187/02); Ministerio de Ecología y Recursos Naturales Renovables (MEyRNR, 048/2013, 072/2014, 061/2015, 073/2016, 035/2017, 047/2018 and 005/2019), Programa de Recursos Naturales y Medio Ambiente (PRNyMA, 01/2016); Brazil, Instituto Chico Mendes de Conservação da Biodiversidade (ICMBio, Sistema de Autorização e Informação em Biodiversidade SISBIO, 12920–3). Ecuador, Ministerio del Ambiente Ecuador 011-2018-IC-FAU-DNB/MA.

**Fig 1:**
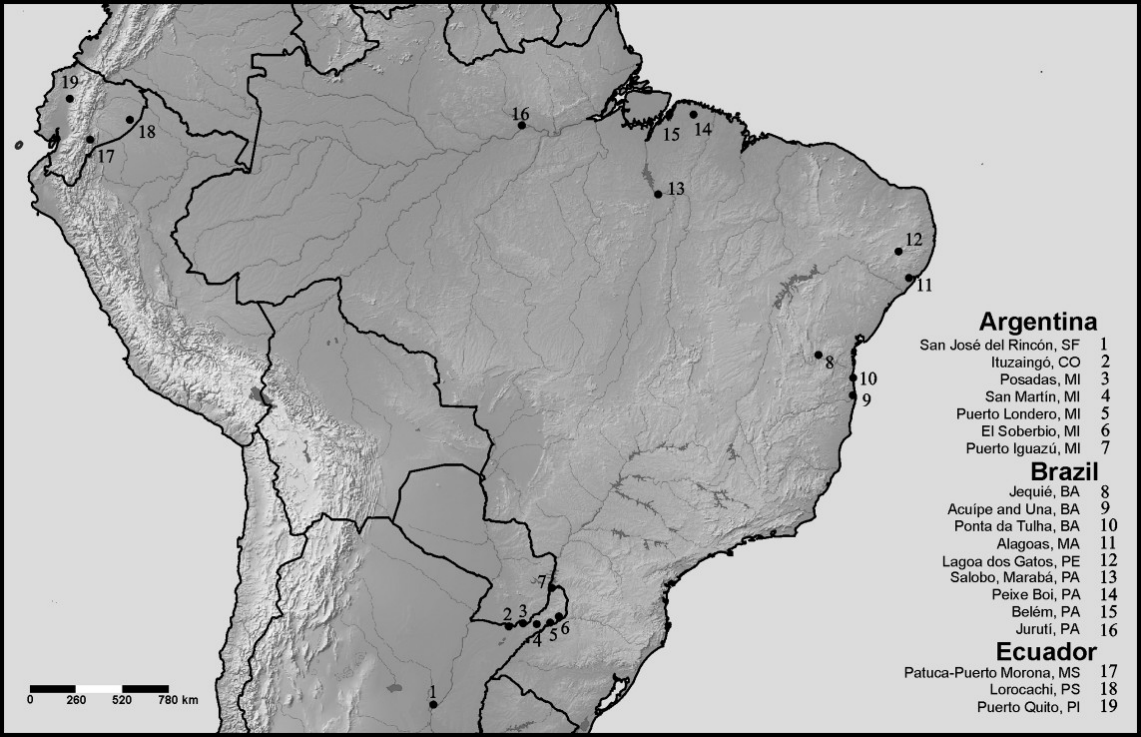
Map of South America showing the collecting localities of the species surveyed in the present study. Santa Fe (SF), Corrientes (CO), Misiones (MI), Bahia (BA), Maceió (MA), Pernambuco (PE), Pará (PA), Morona Santiago (MS), Pastaza (PS), Pichincha (PI). For additional voucher information see the S1 File. The map was created using SimpleMappr (https://www.simplemappr.net), an online tool to produce publication-quality point maps licensed under CC0 1.0 (Public Domain Dedication).

The karyotypes of 16 species are studied here for the first time (*Dryaderces pearsoni*, *Nyctimantis arapapa*, *N*. *rugiceps*, *Osteocephalus buckleyi*, *O*. *fuscifacies*, *O*. *oophagus*, *O*. *planiceps*, *O*. *leprieurii*, *O*. *taurinus*, *Osteopilus vastus*, *Phyllodytes gyrinaethes*, *P*. *melanomystax*, *P*. *praeceptor*, *Trachycephalus helioi*, *T*. *dibernardoi*, and *T*. *jordani*). Locality data of each specimen and the cytogenetic techniques employed are indicated in Fig 1 and Table 1, respectively. Additional information for each sample is given in the S1 File. For *O*. *taurinus*, we followed the phylogenetic hypothesis of Jungfer et al. [11], considering the specimens analyzed in this study as *O*. *taurinus sensu stricto*, and those previously studied from French Guiana by Anderson [19] as *O*. *taurinus* candidate species 5 [11]. Moreover, because Schmid et al. [18] did not indicate the collecting site of the specimen from Venezuela studied by them, we could not determine the precise taxonomic status of that sample as there occur three candidate species phylogenetically related to *O*. *taurinus* in this country (*O*. *taurinus* candidate species 2, 3 and 5, [11]).

**Table 1:** Studied species and the cytogenetic techniques applied for each one.

Genus	Species	Locality	N	Differential Techniques
*Dryaderces*	*D*. *piersoni*	**Brazil.** Pará, Jurutí, Mutúm.	1♀	Ag-NORs, C-bands, DAPI/CMA_3_, rDNA
*Itapotihyla*	*I*. *langsdorffii*	**Argentina.** Misiones, Iguazú, Puerto Iguazú.	1♂	Ag-NORs, C-bands, rDNA
*Nyctimantis*	*N*. *arapapa*	**Brazil.** Bahia, Jequié; Ilhéus, Acuípe.	1♂ 1♀	Ag-NORs, C-bands, DAPI/CMA_3_, rDNA, telDNA
	*N*. *rugiceps*	**Ecuador.** Pastaza, Lorocachi.	1♂	Ag-NORs, C-bands, DAPI/CMA_3_, rDNA
	*N*. *siemersi*	**Argentina.** Corrientes, Ituzaingó.	2♂ 1♀	Ag-NORs, C-bands, DAPI/CMA_3_, rDNA
*Osteocephalus*	*O*. *buckleyi*	**Brazil.** Pará, Jurutí, Acampamento Barroso; Mutúm.	2♂ 1♀	Ag-NORs, C-bands, DAPI/CMA_3_, rDNA, telDNA
	*O*. *fuscifacies*	**Ecuador.** Morona Santiago, Gualaquiza, Bomboiza.	1♂ 1♀	Ag-NORs
	*O*. *leprieiurii*	**Brazil.** Pará, Jurutí, Mutúm; Marabá, Salobo.	2♂	C-bands
	*O*. *oophagus*	**Brazil.** Pará, Jurutí, Acampamento Barroso.	1u	Ag-NORs, C-bands, DAPI/CMA_3_, rDNA
	*O*. *planiceps*	**Ecuador.** Morona Santiago, Vía Patuca-Puerto Morona.	1j	Ag-NORs, C-bands, DAPI/CMA_3_, rDNA, telDNA
	*O*. *taurinus*	**Brazil.** Pará, Jurutí, Acampamento Barroso; Capiranga Plateau.	2♂ 1u	Ag-NORs, C-bands, DAPI/CMA_3_, rDNA, telDNA
*Osteopilus*	*Os*. *septentrionalis*	Pet trade.	3u	Ag-NORs, C-bands, DAPI/CMA_3_, rDNA
	*Os*. *vastus*	Pet trade.	4u	Ag-NORs, C-bands, DAPI/CMA_3_, rDNA
*Phyllodytes*	*P*. *edelmoi*	**Brazil.** Alagoas, Maceio.	1♂	Ag-NORs, C-bands, DAPI/CMA_3_, rDNA, telDNA
	*P*. *gyrinaethes*	**Brazil.** Pernambuco, Lagoa dos Gatos, RPPN Pedra D'Antas.	2♀ 1♂	Ag-NORs, DAPI/CMA_3_, rDNA, telDNA
	*P*. *melanomystax*	**Brazil.** Bahia, Ilhéus, Ponta da Tulha.	1♂	Ag-NORs, telDNA
	*P*. *praeceptor*	**Brazil.** Bahia, Una.	1u	Ag-NORs, DAPI/CMA_3_
*Trachycephalus*	*T*. *dibernardoi*	**Argentina.** Misiones, Guaraní, El Soberbio; 25 de Mayo, Puerto Londero.	1♀ 1♂	Ag-NORs, C-bands, DAPI/CMA_3_, rDNA
* *	*T*. *helioi*	**Brazil.** Pará, Jurutí, Acampamento Barroso.	2♂	Ag-NORs, C-bands, DAPI/CMA_3_, rDNA
	*T*. *jordani*	**Ecuador.** Pichincha, Puerto Quito.	1u	Ag-NORs, C-bands, DAPI/CMA_3_, rDNA
	*T*. *typhonius*	**Brazil.** Pará, Peixe Boi; Belém; **Argentina.** Misiones, Oberá, San Martín; Iguazú, Puerto Iguazú; Capital, Posadas; Santa Fé, Capital, near San José del Rincón.	3♀ 8♂ 1u	Ag-NORs, C-bands, rDNA

N = Number of specimens analyzed. j = juvenile, u = undetermined.

Mitotic chromosome preparations were obtained from bone marrow and intestinal epithelium [24] and stained with 5% buffered Giemsa solution or submitted to differential staining methods. We performed silver staining to detect the location of the active nucleolar organizer regions or Ag-NORs [25] and C-bands to evidence constitutive heterochromatin [26]. Fluorescence *in situ* hybridization (FISH) was carried out on mitotic preparations following Pinkel et al. [27] protocol: biotin-labeled 18S rDNA probes (BioNick DNA Labeling System, Invitrogen) were detected with avidin-Cy3 or avidin-FITC, and telomeric regions were revealed using all-human telomere digoxigenin-labeled probes (TTAGGG)n (Oncor P4097-DG5), following manufacturer's protocol and detected with anti-dig-FICT (or anti-dig-Cy3). The two base-specific fluorochromes DAPI (4',6-diamino-2-phenylindole) and CMA_3_ (chromomycin A3) were used following Schweizer and Ambros [28] after denatured preparations with FISH procedure following the modifications of Barros e Silva and Guerra [29]. Chromosomes of *Os*. *septentrionalis* and *Os*. *vastus* were obtained from cultured lymphocyte cells that were treated with BrdU after Wiley et al. [30] and Wiley and Little [31]. We used the terminology proposed by White [32], considering n as the gametic or haploid chromosome number, 2n as the somatic chromosome number, and FN as the fundamental number (i.e., the total number of chromosome arms per mitotic cell). Because all the karyotyped species in Lophyohylini and almost all Hylidae are diploid, we refer to the gametic number (n) and basic number (x) as equals.

The relative length of mitotic chromosomes and their centromeric index (CI) were obtained using the software Micromeasure 3.3 [33], terming short and long chromosome arms as p and q, respectively. Chromosome morphology was classified as metacentric, submetacentric, subtelocentric, and telocentric, as suggested by Green and Sessions [34].

We optimized the haploid chromosome number and NOR position among the lophyohylines (see the S1 Table for chromosome information in Lophyohylini) on the phylogenetic hypothesis of Blotto et al. [3], following the considerations exposed by Ferro et al. [17]. We considered the character states n = 11, 12, 14, and 17; and NORs in pairs 2, 5, 7, 9, 11, and 17. Optimizations were done with TNT v1.1 [35], considering the states of both characters as unordered transformations.

## Results

**Fig 2:**
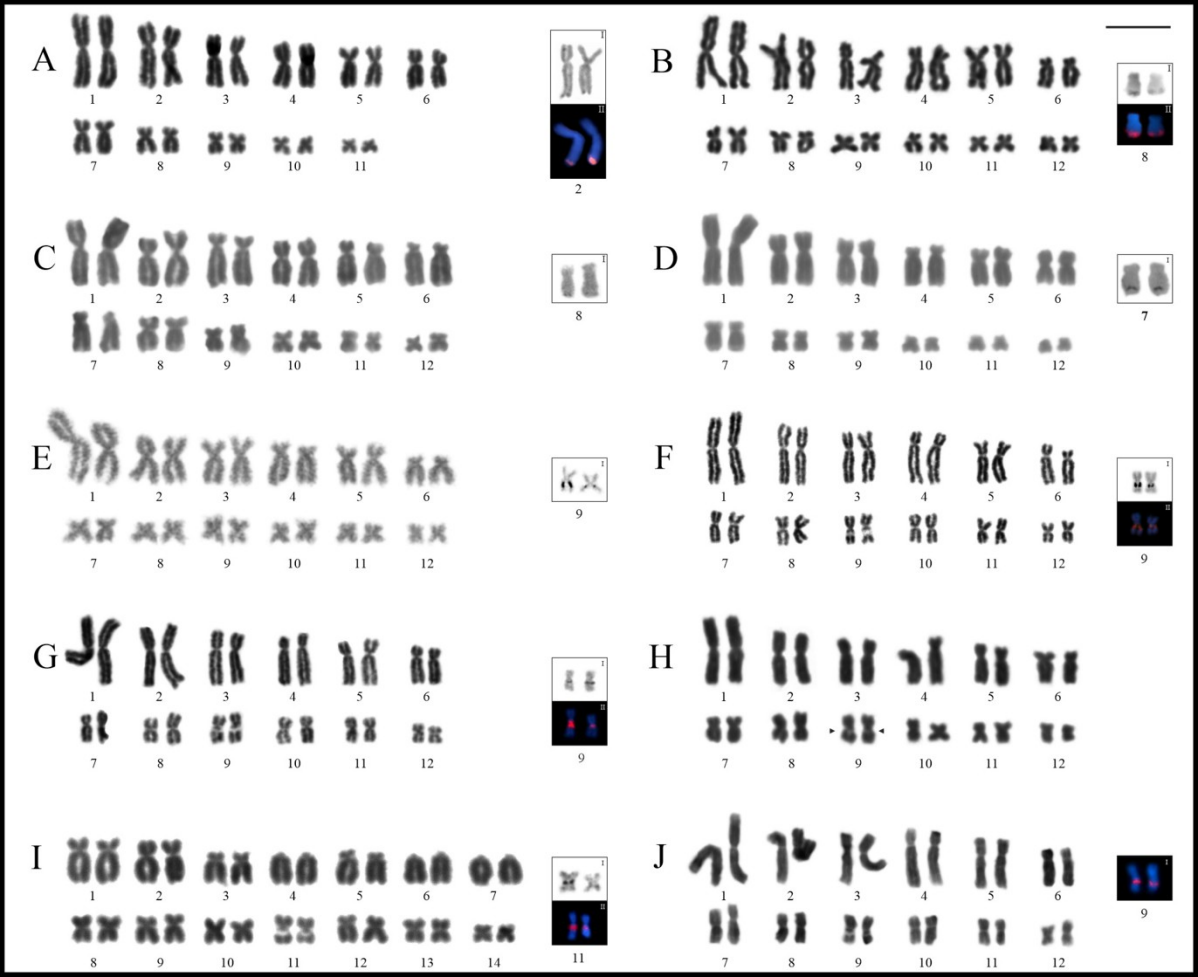
Giemsa stained karyotypes of *Phyllodytes* and *Osteocephalus*. **A.** *Phyllodytes edelmoi*. **B.** *P*. *gyrinaethes*. **C.** *P*. *praeceptor*. **D.** *P*. *melanomystax*. **E.** *Osteocephalus fuscifacies*. **F.** *O*. *taurinus*. **G.** *O*. *oophagus*. **H.** *O*. *leprieurii*. **I.** *O*. *buckleyi*. **J.** *O*. *planiceps*. Squares show chromosomes bearing the NORs after silver staining (**I**) and with FISH using a 18S DNA probe (**II**).

Most species showed karyotypes with 2n = 24, excepting *Phyllodytes edelmoi* and *Osteocephalus buckleyi* that had 2n = 22 and 28, respectively (Fig 2). The morphology of chromosomes of each species is detailed in the S2 Table. In general, karyotypes had a single pair of homologues with NOR sites, in most cases associated with secondary constrictions, always staining CMA_3_^+^ (DAPI^–^) and showing bright hybridization signals with the 18S probe. In all studied species, heterochromatin was remarkably scarce, and C-bands were associated with the NOR sites or had centromeric distribution. In the latter situation, almost all species showed centromeric marks positive for the fluorochrome CMA_3_ (S1 Fig). See the S1 Table for a summary of cytogenetic information for Lophyohylini.

**Fig 3:**
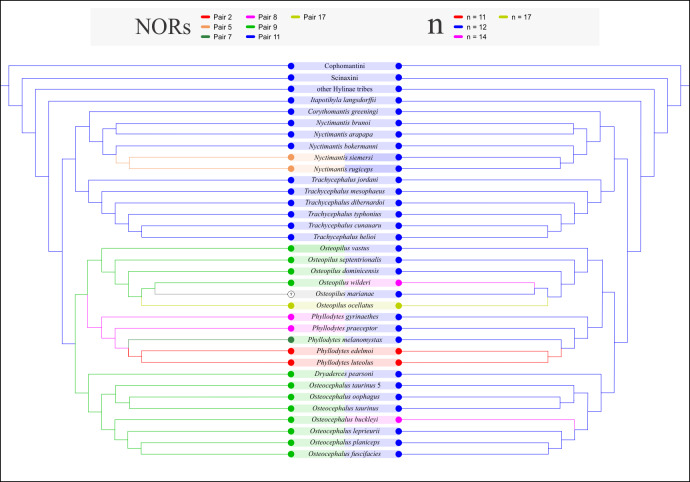
Ancestral character state reconstruction of the position of NORs (left) and the haploid number (right) in Lophyohylini on a condensed tree from the phylogenetic hypothesis of Blotto et al. [3]. For the complete optimizations including all taxa see S2 Fig.

The ancestral character state reconstruction of both characters, the haploid number and NORs, on the phylogenetic hypothesis of Blotto et al. [3] are shown in Fig 3 and S2 Fig. The haploid chromosome number of n = 12 was recovered as the plesiomorphic state of Lophyohylini and n = 14 is an autapomorphy of *Osteopilus wilderi*. Besides, a NOR on pair 11 is plesiomorphic for the tribe, whereas NORs on pairs 8 and 9 optimized as synapomorphies of a subclade of *Phyllodytes*, and the clade including *Osteopilus*, *Phyllodytes*, *Tepuihyla*, *Dryaderces*, and *Osteocephalus*, respectively. Other transformations for both characters are discussed below.

### Phyllodytes

*Phyllodytes gyrinaethes*, *P*. *melanomystax*, and *P*. *praeceptor* had karyotypes with 2n = 24 (FN = 48), whereas *P*. *edelmoi* had 2n = 22 (FN = 44) (Fig 2A–2D). The NOR sites were located distally on pairs 2q in *P*. *edelmoi* (Fig 2A), 8q in *P*. *gyrinaethes* and *P*. *praeceptor* (Fig 2B and 2C), and 7q in *P*. *melanomystax* (Fig 2D), corroborated by FISH in *P*. *edelmoi* and *P*. *gyrinaethes* (Fig 2A and 2B).

**Fig 4:**
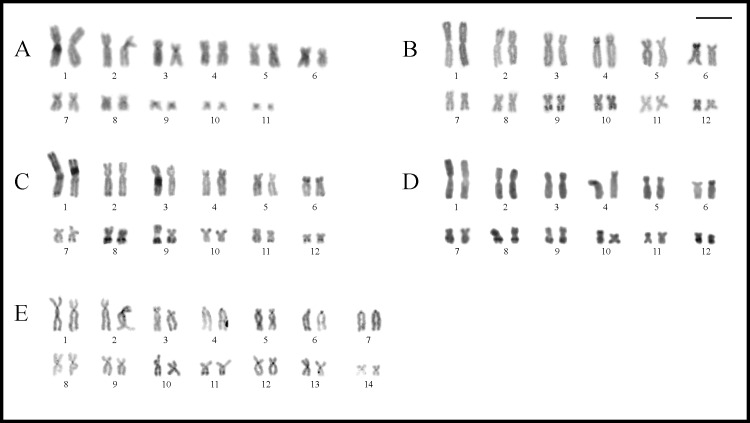
C-banded karyotypes of *Phyllodytes* and *Osteocephalus*. **A.** *Phyllodytes edelmoi*. **B.** *Osteocephalus taurinus*. **C.** *O*. *oophagus*. **D.** *O*. *leprieurii*. **E.** *O*. *buckleyi*.

C-bands were observed on the centromeres of pairs 2–3, 5, and 7‒11 of *Phyllodytes edelmoi* (Fig 4A). Fluorescent CMA_3_^+^ bands, additional to the NOR sites, were detected in the centromeres of *P*. *edelmoi* and *P*. *praeceptor* and, in the latter, also on the long arms of pairs 3–4, and 7 (S1 Fig).

**Fig 5:**
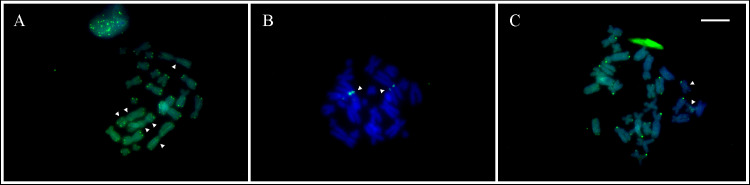
Interstitial telomeric sequences (ITS) in Lophyohylini. Metaphase plates showing ITS detected with FISH with telomeric DNA probe (using FITC fluorochrome). **A.** *Phyllodytes edelmoi*. **B.** *Phyllodytes melanomystax*. **C.** *Osteocephalus buckleyi*. The white arrowheads indicate the ITS. It should be noted that in order to improve the detection of ITS in *P*. *melanomystax*, the distal telomeric signals are not visualized in the metaphase shown in (**B**).

In the three species studied by FISH with telomeric DNA probe, *Phyllodytes edelmoi* (Fig 5A), *P*. *melanomystax* (Fig 5B), and *P*. *gyrinaethes* (S3 Fig), fluorescent signals were detected in the distal region of all chromosomes. Additional interstitial telomeric sites (ITS) were observed in *P*. *edelmoi* and *P*. *melanomystax*, although varying in the intensity and location of the signals. In *P*. *edelmoi* the ITS were present on one arm of pair 1 and both arms of pair 2, whereas in *P*. *melanomystax*, conspicuous pericentromeric ITS were only on one arm of chromosomes of pair 1 (Fig 5A and 5B).

### Osteocephalus

*Osteocephalus fuscifacies*, *O*. *leprieurii*, *O*. *oophagus*, *O*. *planiceps*, and *O*. *taurinus*, shared karyotypes with 2n = 24 with all bi-armed chromosomes (FN = 48), while a 2n = 28 was observed in *O*. *buckleyi*, with pairs 4, 6, and 7 telocentric (FN = 50) (Fig 2E–2J). The NOR sites were located interstitially on 9q in *O*. *fuscifacies* (Fig 2E), *O*. *taurinus* (Fig 2F), *O*. *oophagus* (Fig 2G), and *O*. *planiceps* (Fig 2J), and on pair 11q in *O*. *buckleyi* (Fig 2I). In *O*. *fuscifacies*, the only specimen studied with Ag-NORs (QCAZ 74202), showed them heteromorphic in size. In *O*. *leprieurii*, although it was not possible to detect NORs due to the quality of preparations, conspicuous secondary constrictions were observed in an interstitial position on 9q (Fig 2H).

*Osteocephalus leprieurii*, *O*. *oophagus*, and *O*. *taurinus* showed similar patterns of C-bands, characterized by the presence of conspicuous interstitial and telomeric bands on pairs 6p, 8q, 9q, and 12q (Fig 4B–4D). *Osteocephalus buckleyi* showed C^+^ bands on all chromosome pairs, with additional telomeric bands on pairs 11q and 13p (Fig 4E). In the karyotypes of the four studied species with the fluorochromes DAPI and CMA_3_ (*O*. *buckleyi*, *O*. *oophagus*, *O*. *planiceps*, and *O*. *taurinus*), centromeres were CMA_3_^+^, in addition to NOR sites (S1 Fig).

FISH experiments with the telomeric DNA probes showed distal signals in *Osteocephalus planiceps* and *O*. *taurinus* (S3 Fig), while in *O*. *buckleyi*, an additional ITS was detected in the centromeric region of one of the homologues of pair 12 (Fig 5C).

**Fig 6:**
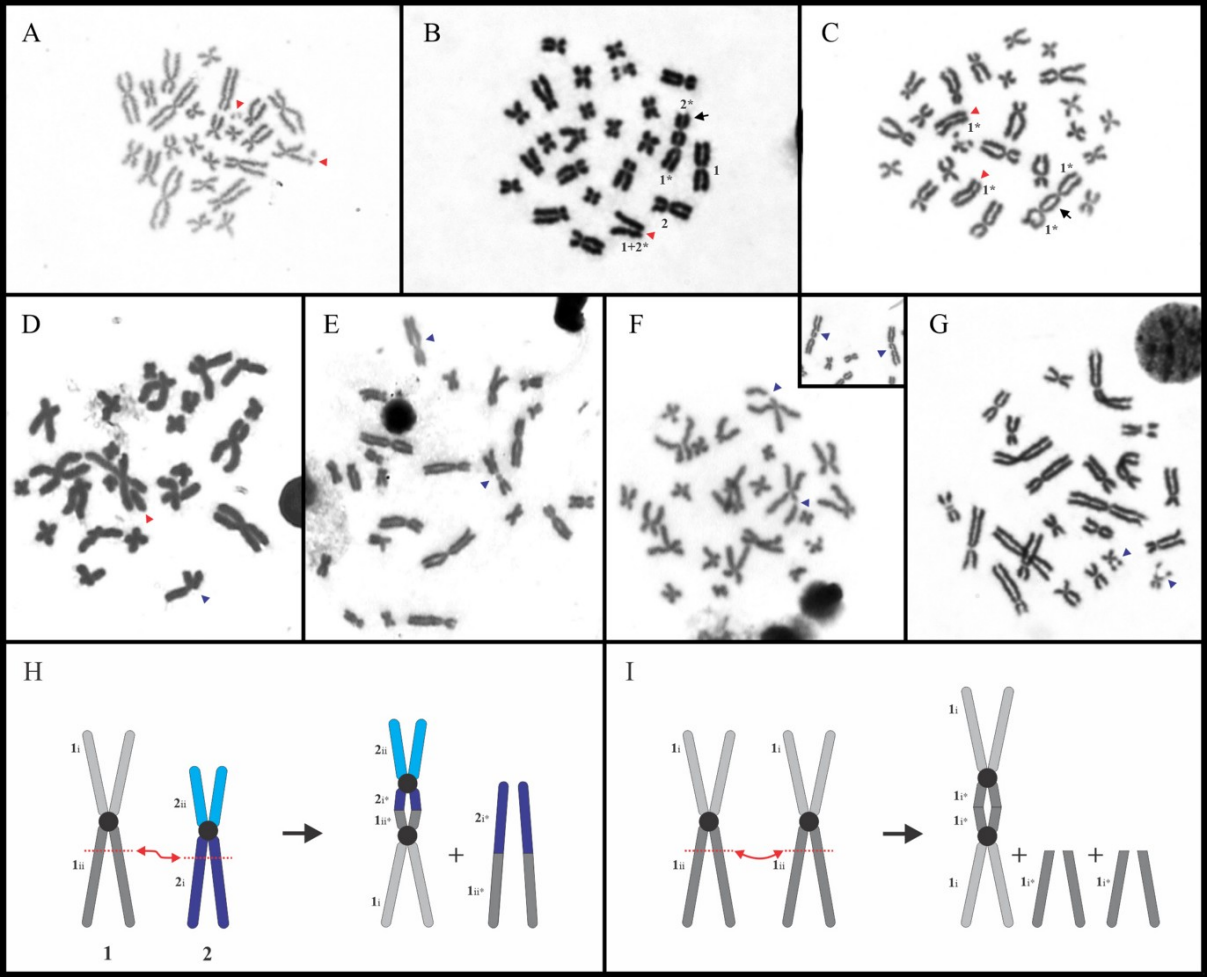
Spontaneous rearrangements and fragile sites in Lophyohylini. **A–C, H, I.** *Osteocephalus taurinus*. **D.** *Nyctimantis siemersi*. **E.** *Trachycephalus typhonius*. **F.** *Osteopilus vastus*. **G.** *Dryaderces pearsoni*. Red arrowheads point chromosome fragments (**A–D**), whereas blue arrowheads indicate the secondary constrictions not associated with the NOR sites (**D–G**). In (**B**) and (**C**), the black arrows show the dicentric chromosomes resulting from spontaneous rearrangements in *O*. *taurinus*, and their respective schematic representation is shown in (**H**) and (**I**). Inset: partial mitotic metaphase of *Os*. *vastus*.

In three metaphase plates of two specimens of *Osteocephalus taurinus* (2 of 22 cells in PS 430, 1 of 10 cells in PS 467) chromosome variation were detected resulting from spontaneous fission and fusion rearrangements (Fig 6A–6C). One cell showed two additional small chromosome fragments, but it was not possible to identify the chromosome/s involved in this phenomenon, as no gross morphological differences were detected in the karyotype (Fig 6A). In the remaining two cells, on the other hand, chromosome fragments and dicentric chromosomes were observed, in which the latter were formed by rearrangements involving non-homologous chromosomes of pairs 1 and 2 (Fig 6B and 6H), or both homologues of pair 1 (Fig 6C and 6I).

### Nyctimantis

**Fig 7:**
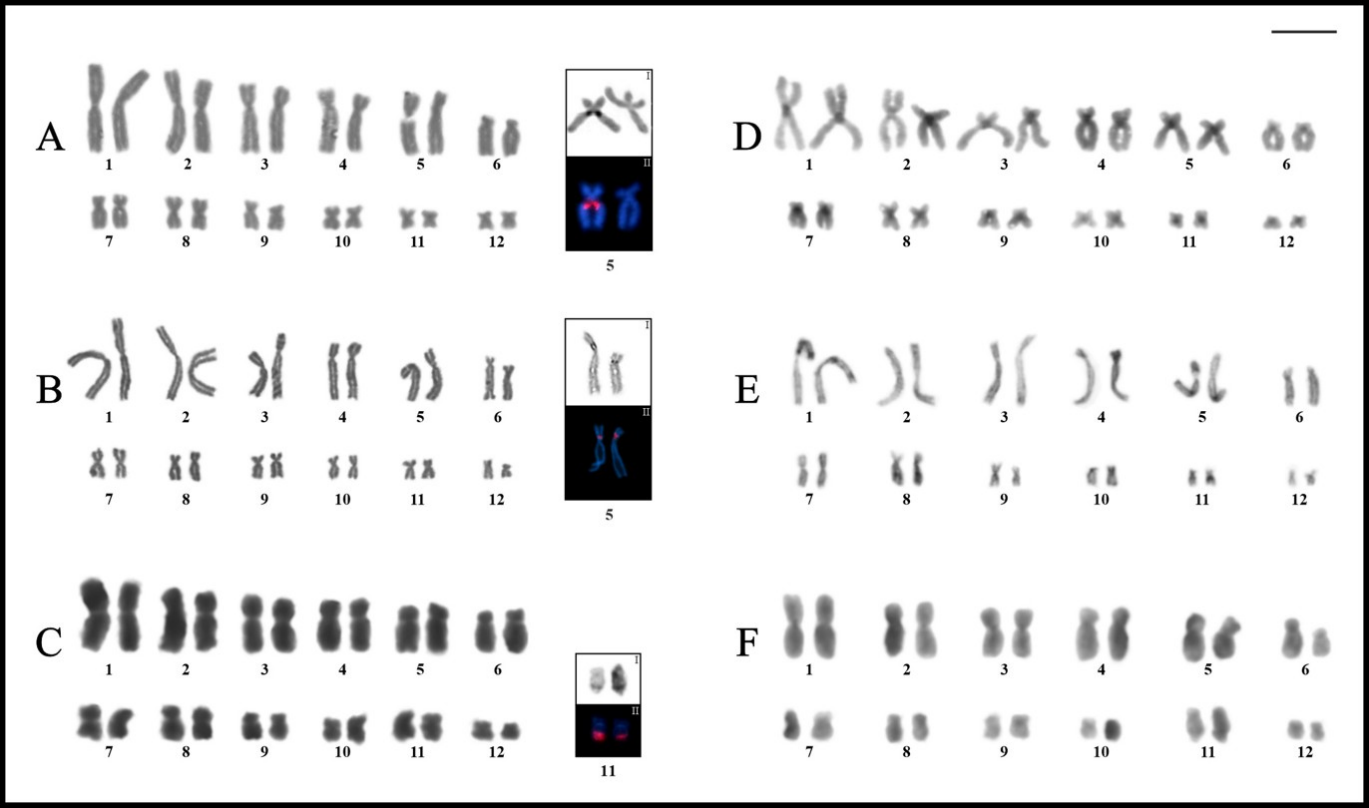
Karyotypes of *Nyctimantis*. **A, D.** *N*. *siemersi*. **B, E.** *N*. *rugiceps*. **C, F.** *N*. *arapapa*. Conventional staining (left) and C-bands (right). The squares show chromosomes carrying NOR sites: **I.** Ag-NORs, **II.** FISH with 18S rDNA.

All *Nyctimantis* analyzed shared karyotypes with 2n = 24 (FN = 48). In *N*. *siemersi*, the three specimens studied (2♂ and 1♀) had a single secondary constriction pericentromeric on 5q. Ag-NORs were studied in the two male specimens (LGE 11192, 11194) and corroborated by FISH in only one of them, LGE 11192 (Fig 7A). In *N*. *rugiceps* the NORs were located pericentromerically on pair 5p (Fig 7B), and in *N*. *arapapa* distally on pair 11q (Fig 7C).

*Nyctimantis siemersi* showed C-bands in the centromeric and pericentromeric regions of all chromosomes (Fig 7D), with CMA_3_^+^ marks on this position (S1 Fig). In *N*. *rugiceps*, heterochromatic bands were restricted to the interstitial regions of chromosome pairs 8q and 11q, and the pericentromeric regions of 10p (Fig 7E). Similarly, in this species, fluorescent CMA_3_^+^ bands were observed on 5p associated with NORs, and the centromeres (S1 Fig). In *N*. *arapapa*, all chromosome pairs showed faint centromeric C-bands (Fig 7F) that were, in addition to the NOR sites, DAPI^–^/CMA_3_^+^ (S1 Fig). In this species, the FISH with the telomeric probe showed positive signals on the distal region of all chromosomes (S3 Fig). In a single cell of one individual of *Nyctimantis siemersi* (LGE 11194), it was possible to detect a chromosomal break on pair 5q, involving one chromatid at the interstitial region of the NOR sites (Fig 6D).

### Trachycephalus

**Fig 8:**
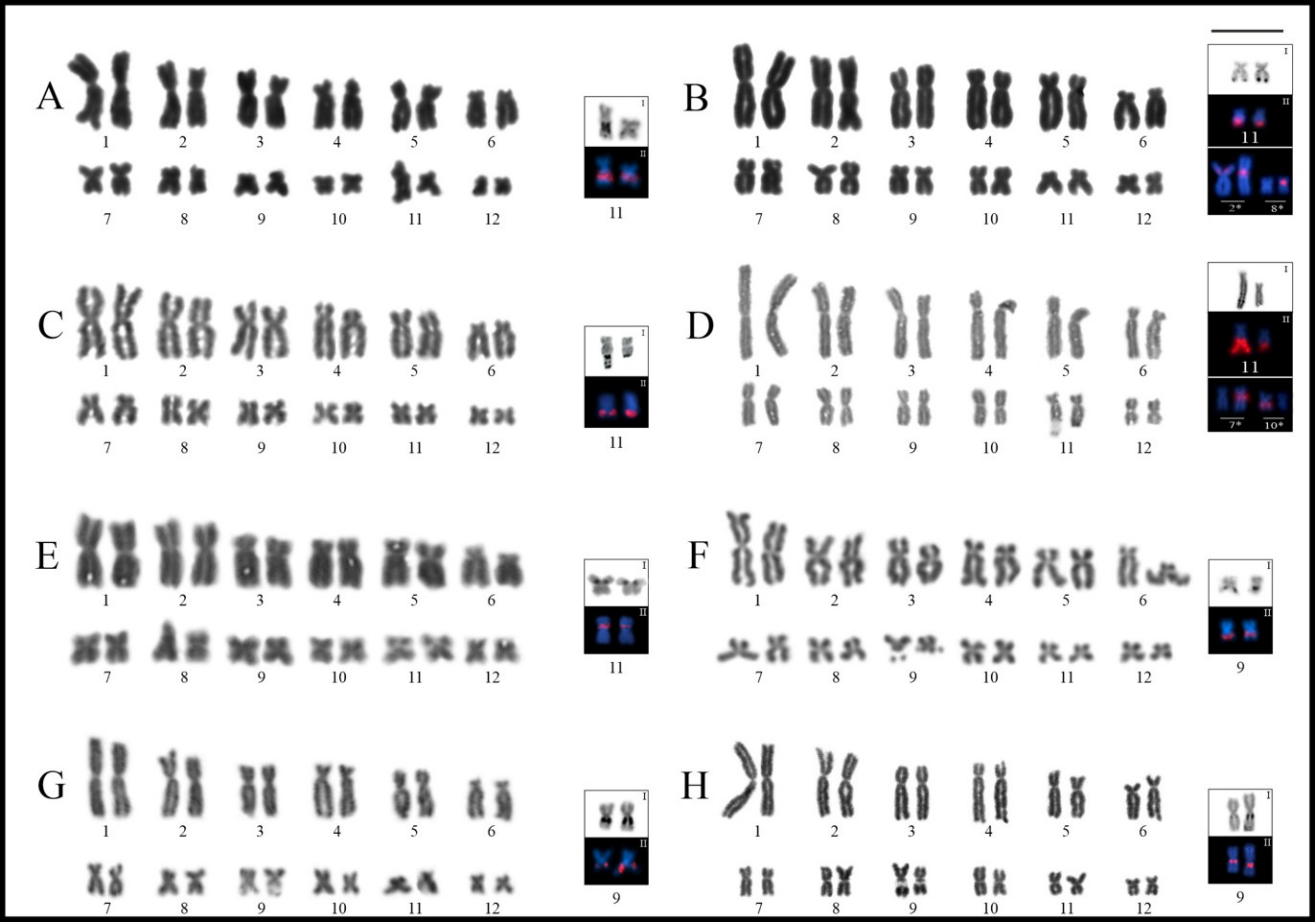
Giemsa stained karyotypes of *Trachycephalus*, *Itapotihyla*, *Osteopilus*, and *Dryaderces*. **A.** *Trachycephalus jordani*. **B.** *T*. *helioi*. **C.** *T*. *dibernardoi*. **D.** *T*. *typhonius*. **E.** *Itapotihyla langsdorffii*. **F.** *Osteopilus septentrionalis*. **G.** *Os*. *vastus*. **H.** *Dryaderces pearsoni*. Squares show chromosomes bearing the NORs after silver staining (**I**) and with FISH using a 18S DNA probe (**II**).

The four analyzed species of this genus had karyotypes with 2n = 24 (FN = 48). The NOR sites were located on 11q, interstitially in *Trachycephalus jordani*, and distally in *T*. *dibernardoi*, *T*. *helioi*, and *T*. *typhonius* (Fig 8A–8D).

**Fig 9:**
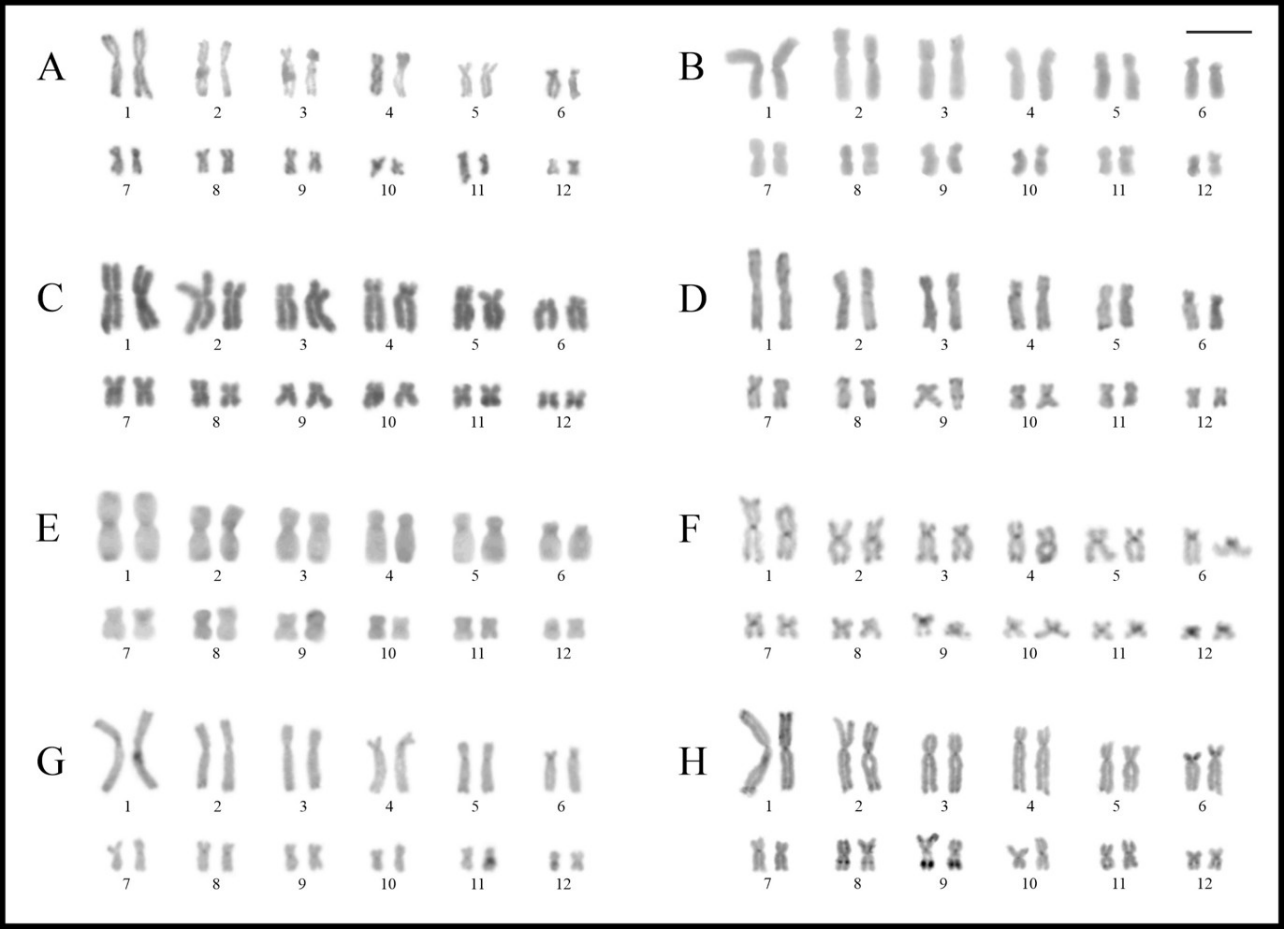
C-banded karyotypes of *Trachycephalus*, *Itapotihyla*, *Osteopilus*, and *Dryaderces*. **A.** *Trachycephalus jordani*. **B.** *T*. *dibernardoi*. **C.** *T*. *helioi*. **D.** *T*. *typhonius*. **E.** *Itapotihyla langsdorffii*. **F.** *Osteopilus septentrionalis*. **G.** *Os*. *vastus*. **H.** *Dryaderces pearsoni*.

C-bands were mainly distributed on the centromeres of all species (Fig 9A–9D), with additional bands observed distally on chromosome pair 1p in *Trachycephalus jordani* (Fig 9A), interstitially on 4p and 12q in *T*. *helioi* (Fig 9C), and distally and interstitially on pairs 8q and 9q in *T*. *typhonius*, respectively (Fig 9D).

*Trachycephalus jordani*, *T*. *dibernardoi*, and *T*. *helioi* showed centromeric DAPI^–^/CMA_3_^+^ marks in all chromosomes, whereas in *T*. *dibernardoi* the centromeres were also DAPI^−^but it was not clear a differential pattern for the CMA_3_ fluorochrome (S1 Fig).

Additional rDNA sites were detected on chromosomes of pairs 2 and 8 in *Trachycephalus helioi* (Fig 8B) and of pairs 7 and 10 in *T*. *typhonius* (Fig 8D), which was observed in one specimen of each species (PS 294 and LGE 18980, respectively). In both cases, the chromosomes involved showed positive signals after FISH but were negative for silver Ag-NOR staining. In *T*. *helioi*, bright hybridization 18S signals were present **(i)** on both chromosomes of pair 2p but differing in their location, interstitially in one of the homologues and pericentromerically in the other; and **(ii)** pericentromerically on one of the homologues of pair 8p. In *T*. *typhonius*, hybridization was observed in only one of the homologues of chromosomes pairs 7q (pericentromerically) and 10q (interstitially).

Another variation regarding the NOR sites was evidenced for the size of Ag-NORs between homologue chromosomes in *Trachycephalus jordani*, *T*. *dibernardoi*, and *T*. *typhonius* (Fig 8A, 8C and 8D). In *T*. *dibernardoi* and *T*. *typhonius*, it was possible to detect triplications and quintuplications respectively in good quality mitotic metaphases stained with Ag-NORs, which was also corroborated by FISH (Fig 8A and 8D). In *T*. *dibernardoi*, on the other hand, this technique was not performed in the specimen that showed the heteromorphism.

Finally, in *Trachycephalus typhonius* pericentromeric secondary constrictions or fragile sites were detected on chromosomes of pair 3q that were not associated with NORs or C-bands (Fig 6E). This feature was found in five specimens of Argentine localities, in both homologues in three samples (homozygosis) or only one chromosome in two of them (heterozygosis).

### Itapotihyla langsdorffii

*Itapotihyla langsdorffii* had a karyotype with 2n = 24 (FN = 48), with NOR sites located pericentromerically on pair 11p (Fig 8E). C-bands were observed on the centromeric regions of all chromosomes and distally on pairs 1–3q (Fig 9E).

### Osteopilus

*Osteopilus septentrionalis* and *Os*. *vastus* shared karyotypes with 2n = 24 (FN = 48), with interstitial NOR sites on pair 9q in both species (Fig 8F and 8G).

The two species had similar patterns of C-bands that were restricted to the centromeres, with additional heterochromatin present in *Osteopilus septentrionalis* distally in the long arm of the largest chromosomes (1–4) (Fig 9F and 9G). DAPI^–^/CMA_3_^+^ fluorescent bands were observed interstitially on Pair 9q in both species, coincident with the NOR sites, in the distal region of most chromosomes of *Os*. *septentrionalis* and in all the centromeres in *Os*. *vastus* (S1 Fig).

In two specimens of *Osteopilus vastus*, a conspicuous interstitial secondary constriction not associated with NOR sites were observed on chromosomes 1p (Fig 6F). Curiously, this secondary constriction always involved only one chromatid of such chromosome. In one specimen (Os.5), it was present on both chromosomes 1 in about half of the analyzed cells (14 of 26 cells), whereas in the other specimen (Os.8), this feature was observed only in one chromosome 1 and more infrequently (3 of 48 cells).

### Dryaderces pearsoni

This species had a karyotype with 2n = 24 (FN = 48) with NORs located interstitially in pair 9q (Fig 8H). Heterochromatin showed a centromeric pattern, although conspicuous C-bands were observed interstitially on pairs 6p, 8q, 9q, and 12q, and distally on 11q (Fig 9H). The centromeres of almost all chromosomes showed DAPI^–^/CMA_3_^+^ bands, in addition to the NOR sites. Both homologues of pair 12q exhibited an extra interstitial secondary constriction not associated with NORs composed of rich DAPI^+^/ CMA_3_^–^ heterochromatin (Fig 6G, S1 Fig).

## Discussion

Cytogenetic information available for Lophyohylini currently comprises data on 31 karyotyped species of the 87 species (36%) of the tribe, showing a broad diversity in several chromosomal characters. Different haploid numbers were reported for the tribe, although karyotypes with n = 12 that are composed of all bi-armed chromosomes (FN = 48) are common features present in all studied genera, and represent the plesiomorphic states for n and FN, respectively [18, this study]. Reductions were recorded in *Phyllodytes edelmoi* and *P*. *luteolus* (n = 11, 2n = 22, FN = 44), while the opposite, increments were observed in *Osteocephalus buckleyi* (n = 14, 2n = 28, FN = 50), *Osteopilus wilderi* (n = 14, 2n = 28, FN = 52), and *Os*. *ocellatus* (n = 17, 2n = 34, FN = 48).

*Tepuihyla* and the monotypic genus *Phytotriades* (*P*. *auratus*) remain the only two genera of Lophyohylini lacking any cytogenetic information. However, according to different phylogenetic hypotheses available for the tribe [3,6,9], whatever haploid and (or) fundamental numbers are present in these taxa, the plesiomorphic character states for Lophyohylini, n = 12 and FN = 48 remain unchanged.

It was suggested that within Lophyohylini (except *Osteocephalus taurinus* candidate species 5), there is a remarkable discontinuity in the size of the first 5 chromosome pairs and the remaining 7 [4, 21]. However, this feature is not evident when comparing differences between the size percentage of the haploid set of pairs 5 and 6 in karyotypes of lophyohylines with 2n = 24 (S3 Table).

### Interstitial telomeric sequences in Lophyohylini

Interstitial telomeric DNA sequences or ITS is a frequent phenomenon observed in vertebrates, and may be the evolutionary consequence of chromosome rearrangements (e.g., inversions or fusions), but also of a variety of molecular mechanisms involving transposition and amplification of telomeric DNA sequences [24,36,37]. Among anurans, the presence of ITS has been reported in 45 species (see [24,38, for reviews, this study]).

The ITS have rarely been related to taxa that have undergone a process of chromosome reduction. However, it is possible to find this association in a few species of *Aplastodiscus* and *Scarthyla goinorum* (Hylidae), and *Leptodactylus* aff. *podicipinus* (Leptodactylidae). In *Aplastodiscus*, two independent reductions changed the plesiomorphic haploid number of n = 12 to n = 11 in the species of the *A*. *albofrenatus* group, and to n = 10 and n = 9 in the species of the *A*. *albosignatus* group [39–41]. It was stated that the ITS reported in *Aplastodiscus* may not be directly related to the chromosome reductions observed in the genus [40]. However, it should be noted that ITSs were only detected in species with reduced karyotypes and not in *A*. *perviridis*, the only taxon with n = 12 studied by FISH with a telomeric DNA probe. Similarly, *Scarthyla goinorum* is the only species within Pseudini that has a reduced karyotype (n = 11), in addition to the presence of ITS on chromosomes of pair 3 [15]. Based on these two facts, Suárez et al. [15] proposed that the ITS in this species would be directly related to karyotype reduction. It must be noticed at this point that only one additional species of the tribe with a non-reduced karyotype (n = 12) had been studied by FISH (i.e., *Lysapsus laevis*). In *Leptodactylus* sp. aff. *podicipinus* (n = 10, 2n = 20), ITS are present in the centromeric region of a small pair of chromosomes, but centromeric ITS were also confirmed in *L*. *podicipinus*, a phylogenetically related species that shows the plesiomorphic karyotype of Leptodactylidae (n = 11, 2n = 22, [42]).

In 14 species of lophyohylines telomeric DNA was mapped by FISH, and ITS were present in 4 of them: *Itapotihyla langsdorffii*, *Phyllodytes edelmoi*, *P*. *melanomystax*, and *Osteocephalus buckleyi* [19,22, this study]. In a similar way to what is observed in almost all other species of anurans, the heterochromatic ITS (het-ITS) pattern observed in the karyotype of *I*. *langsdorffii* represents an apomorphic condition, which is probably related to dispersion and amplification mechanisms responsible for the internalization of telomeric-like DNA sequences [22].

Gruber et al. [22] reported ITS in *Phyllodytes edelmoi*, later described as het-ITSs by Schmid and Steinlein [38] and Schmid et al. [18]. However, it should be mentioned that according to the original description, no C-bands were detected for this species [22]. Indeed, we did not detect for this species positive C-bands apart from those of the centromeres. The similar gross morphology of pairs 1 and 2 between the studied species of *Phyllodytes* [22, this study] and the presence of ITS in chromosomes of pair 2 in *P*. *melanomystax*, suggest that these pairs would not have participated in the karyotype reduction of the genus (from 24 to 22) and that ITS would have an earlier evolutionary origin. Nonetheless, we cannot discard further rearrangements involving these pairs of chromosomes (see **NOR sites and their phylogenetic information in Lophyohylini**). The absence of ITS signals in *P*. *gyrinaethes* provides evidence to propose that the ITS of chromosome pair 2 may represent a putative synapomorphy of an internal clade of *Phyllodytes*, comprising species with 2n = 22 and 2n = 24. However, a better sampling is necessary since only 5 of 14 species of the genus were karyotyped, and only three of them studied by FISH with telomeric DNA.

Among six studied species of *Osteocephalus* [19, this study] there is a plesiomorphic karyotype excepting *O*. *buckleyi* (n = 14, 2n = 28, FN = 50). Different sources of evidence (i.e., C-bands, NORs, chromosome size, and morphology) provide further support that the six minor pairs are conserved in the clade *Osteocephalus* + *Dryaderces* (see below). The presence of pericentromeric ITS on both homologues of pair 13 in *O*. *buckleyi* would not be associated with the chromosome reorganization observed in this species, and possibly originated by different mechanisms.

### Fragile sites and their possible role in the chromosomal evolution of Lophyohylini

The secondary constrictions or chromosome gaps are usually the consequence of chromosome regions that are associated with the NORs sites, although they can also be formed by constitutive heterochromatin or fragile sites [24]. Fragile sites are defined as specific chromosomal regions that are prone to break and participate in chromosome rearrangements. Unlike anurans, this feature has been extensively studied in humans, and depending on their prevalence in a population, they can be defined as rare or common fragile sites (see [43] for a review).

As in other groups of anurans, the occurrence of spontaneous chromosome rearrangements in natural populations of Hylidae is extraordinarily infrequent and, as far as we know, only two isolated cases have been documented in addition to the present study. Feitosa et al. [44] studied the effects of naturally occurring radiation in the frequency of chromosomal rearrangements in populations of *Aplastodiscus perviridis* and *Boana albopunctata* from Morro do Ferro (Minas Gerais, Brazil). Additionally, Anderson [19] described chromosomal abnormalities in the two genera of lophyohylines *Osteocephalus* and *Osteopilus*.

In this work, we found variation in several species of Lophyohylini regarding secondary constrictions not associated with NORs in *Dryaderces pearsoni*, *Osteopilus vastus*, and *Trachycephalus typhonius*; spontaneous chromosome rearrangements in *Nyctimantis siemersi* and *Osteocephalus taurinus*; and NORs site polymorphisms in *N*. *siemersi* and some species of *Trachycephalus*. Although we did not systematically evaluate this variation because it was not our main objective, the frequency of occurrence of this rearrangement is remarkable compared to other anuran groups and deserves to be discussed.

Anderson [19] highlighted that in specimens of *Osteocephalus taurinus* candidate species 5 from French Guiana (as *O*. *taurinus*), there were fragile interstitial sites in coincidence with heterochromatic regions in medium-sized and small chromosomes (pairs 6 and 9). We could also infer the presence of fragile sites in *O*. *taurinus*, by the occurrence of spontaneous rearrangements that generated dicentric chromosomes and chromosome fragments but involving the first two chromosomes pairs. Although almost all interstitial heterochromatic bands previously described by Anderson [19] (inferred from the ideogram figure), were not detected in the present study for *O*. *taurinus*, it is tempting to propose that there is co-location between the putative sites where such chromosome breakpoints occurred in *O*. *taurinus* and those interstitial heterochromatic bands of *O*. *taurinus* candidate species 5.

A chromatid gap or secondary constriction, not associated with NORs or heterochromatin, was observed in two specimens of *Osteopilus vastus*: present on a single chromatid of only one (heterozygosis) or the two chromosomes of pair 1 (homozygosis). As stated, our cell preparations of *Osteopilus* were obtained from lymphocyte cultures submitted to a BrdU treatment. Although we do not have an explanation about this rare feature, it is interesting to note that all records in both specimens occurred on precisely the same chromosome region. Under certain conditions, fragile sites can be induced at a low frequency as single chromatid gaps or breaks in cultured cells [43]. Although this could be a possible cause for this extraordinary phenomenon, this was never mentioned in similar studies performing BrdU induction in anurans (e.g., [14,21,22,30,31,45–49]). Interestingly, the remarkable similarity between chromosomes 1 and 2 of *Os*. *ocellatus* and *Os*. *wilderi*, respectively [18,19,23], resembles the morphology of chromosome 1 of *Os*. *vastus* if we consider a chromosome fission at that site, which would suggest that the putative fragile site observed in *Os*. *vastus* could have played a role in the chromosomal evolution of the genus.

Almost all species of *Osteopilus* share the plesiomorphic karyotype number, with the exceptions of *Os*. *wilderi* [19] and *Os*. *ocellatus* [18,19,23]. The different diploid numbers present in these two species could be explained by the occurrence of translocations involving whole-arms (i.e., Robertsonian translocations), although other rearrangements could play a role in the change of the FN from 48 to 52 observed in *Os*. *wilderi*. The phylogenetic relationships of *Osteopilus* [3,9] suggest independent origins for the changes observed in *Os*. *ocellatus* and *Os*. *wilderi*. However, it must be noticed that the karyotype of *Os*. *marianae* (inferred from the ideogram representation in [19]), differs in chromosome morphology and size from that of other species of the genus with 2n = 24 chromosomes, particularly regarding pairs 4 and 5. It is possible that the karyotype of *Os*. *marianae* could have had additional rearrangements that have led to mimic the plesiomorphic state. In this sense, it is essential to study the cytogenetics of *Os*. *crucialis*, the sister species of *Os*. *ocellatus*, and also perform differential cytogenetic techniques in *Os*. *marianae*.

### NOR sites and phylogenetic information

Within Lophyohylini, most species show a single pair of NORs located on small pairs of chromosomes with similar morphology [18, this study]. This condition is also present in Cophomantini [17], Scinaxini [13,16], and Hylini [18], suggesting a putative homeology between the chromosomes carrying this marker [13,14]. Moreover, the BrdU replication banding pattern information gathered from different neotropical species of hylids strengthens this hypothesis [21, 49].

Like other groups of Hylidae, the NORs of lophyohylines show interesting variation. First, their presence in chromosomes of pair 11 that is the plesiomorphic condition, observed in almost all species of the major clade comprising *Trachycephalus*, *Corythomantis*, and *Nyctimantis*, which is also shared by the basal monotypic genus *Itapotihyla* (i.e., *I*. *langsdorffii*) [21,22,50, this study]; in this context, it would be the most parsimonious condition expected for its sister taxon *Phytotriades auratus*. In *Nyctimantis*, there are two patterns for the chromosome location of the NORs. In *N*. *arapapa*, *N*. *bokermanni*, and *N*. *brunoi*, they are terminal on pair 11q (as pair 10 in [21,22], this study), whereas in *N*. *siemersi* and *N*. *rugiceps* they are pericentromeric on chromosome pair 5 [20, this study]. The NORs on pair 5 observed in *N*. *rugiceps* and *N*. *siemersi*, would likely have a common origin and are a putative synapomorphy of a less inclusive clade within *Nyctimantis* that includes these species. The differences in the intrachromosomal position of this marker between them, on 5p and 5q respectively, would possibly be the consequence of subsequent modifications (e.g., pericentric inversion). Indeed, this assumption is not unreasonable since both taxa are closely related according to several phylogenetic studies [3,6–9,51,52]. However, to test this hypothesis, it is still necessary to study *N*. *galeata* and *N*. *pombali* included in this clade, and are most closely related to *N*. *rugiceps*.

Three genera of the clade composed of *Tepuihyla*, *Osteocephalus*, *Dryaderces*, *Osteopilus*, and *Phyllodytes* [3] share NOR sites localized interstitially on a small-sized metacentric pair of chromosomes (i.e., pair 9 [19, this study]), differing significantly in size from the pair 11 observed in other lophyohylines. The NORs on pair 9 are found in species of *Osteocephalus* (*O*. *taurinus* candidate species 5, *O*. *taurinus*, *O*. *oophagus*, *O*. *planiceps*, and *O*. *leprieurii* inferred in this study by the presence of secondary constrictions), *Dryaderces* (*D*. *pearsoni*), and *Osteopilus* (*Os*. *dominicensis*, *Os*. *septentrionalis*, and *Os*. *vastus*). Similarly, in *Osteocephalus buckleyi* and *Os*. *wilderi*, despite having a higher number of chromosomes and NORs on pairs 11 and 9, respectively [19, this study], the similarity between the chromosomes carrying NORs in these species and those with 2n = 24 also suggest homeology of these elements. In this way, NORs on pair 9 represents a putative synapomorphy of this poorly supported clade and remains to be studied in *Tepuihyla*. Moreover, an interstitial C-band on pair 8 in the species of *Osteocephalus*, including the observations in *O*. *buckleyi* (on pair 10), and in *Dryaderces pearsoni* [19, this study], represents a putative synapomorphy for *Dryaderces* + *Osteocephalus*.

The NORs occur on pair 2 in *Phyllodytes edelmoi* and *P*. *luteolus* [22, this study], pair 7 in *P*. *melanomystax*, and pair 8 in *P*. *gyrinaethes* and *P*. *praeceptor* (this study). This variation and additional chromosomal differences (i.e., the morphology of pairs 4, 6, and 7) suggest that cytogenetics is a promising source of information for the systematics of *Phyllodytes*; however, its phylogenetic interpretation is sharply limited by the still sparse and scattered cytogenetic sampling of the genus.

### Polymorphisms for the NOR sites in Lophyohylini

In anurans, polymorphisms for the location and size of the NORs are frequent, and in about half of the reported cases have been observed with *in situ* detection with rDNA in addition to Ag-NOR staining [24,30,39,50,53–65]. This marker was studied in almost all lophyohylines showing an impressive variation (S1 Table).

In *Osteopilus septentrionalis*, intra and interchromosomal differences of NORs location were reported (i.e., interstitial on 9q), due to paracentric inversions of pair 9 and to a reciprocal translocation in heterozygosis involving pairs 6 and 9, respectively [19]. Although such variation was not observed in other studies [18,23,66, this study], in the figures provided by Schmid [66] and Schmid et al. [18] for this species (Fig 8C and 571, respectively), the Ag-NORs marks are located in a pericentromeric position. Moreover, multiple Ag-NORs were described in *Os*. *ocellatus* [18,19], supporting the idea that the NORs in *Osteopilus* are highly variable.

Morand and Hernando [20] studied the Ag-NORs in three females of *Nyctimantis siemersi*, reporting that 60% of the cells had NORs on one or two chromosomes of pair 5, while the remaining 40% had them interstitially on one homologue of pair 1. In our study, Ag-NORs were observed in only one chromosome of pair 5 in two males, corroborated by FISH in one of them, and inferred by the presence of remarkable secondary constriction in a female. A similar feature was reported in the lophyohylines *Corythomantis greeningi* and *Nyctimantis brunoi*, although, in these species, the occurrence of a single Ag-NORs was rejected by FISH [21]. The complete deletion of the NOR sites, as it is observed in *N*. *siemersi*, is infrequent among anurans ([66]; see [24] for a review), being difficult to understand its high prevalence without a sex association.

In *Trachycephalus*, four species show conspicuous secondary constrictions associated with Ag-NORs: *T*. *dibernardoi*, *T*. *cunauaru* (as *Trachycephalus* sp. in [18,22]), *T*. *jordani*, and *T*. *typhonius*, which was additionally confirmed by FISH in the last two species [22, this study]. Heteromorphisms for the size of the NORs were observed in other species of lophyohylines: *Corythomantis greeningi*, *Itapotihyla langsdorffii*, *Nyctimantis bokermanni*, *Osteocephalus fuscifacies*, and *P*. *luteolus* [21, this study]. Indeed, the occurrence of variable size between homologs carrying the NOR sites is commonly reported, where unequal crossing between homologues during the first meiotic prophase would change the position of rDNA clusters forming tandem multiplications [66,67].

Finally, the lack of silver impregnation affinity in rDNA positive signals after FISH was confirmed in this study in the two species of *Trachycephalus*, *T*. *helioi* and *T*. *typhonius*, denoting that were transcriptionally inactivated (see [24] for a review). The presence of polymorphic silent NOR sites in Anura is an infrequent feature described only in *Craugastor fitzingeri* [24], *Hyla chrysoscelis*, *H*. *versicolor* [30], and *Scinax tripui* [65]. Although this could be the consequence of sampling bias, as the FISH technique is not routinely used on several specimens to establish a correspondence between the Ag-NOR bands and rDNA sites. Because this feature has so far been poorly studied in this group, it is still premature to draw conclusions about the presence and the position of silenced rDNA sites.

## Conclusions

Among vertebrates, the chromosome evolution of anurans has traditionally been considered to be stable due to the apparent high conservatism of karyotypes of several taxa. However, the recent discovery of significant variation, particularly regarding spontaneous numerical and structural alterations, has started challenging this idea. For instance, Schmid et al. [24,68] described unprecedented rates of spontaneous chromosomal anomalies in several species of direct-developing frogs of the family Hemiphractidae, and particularly of the brachycephaloid families Craugastoridae and Eleutherodactylidae, reaching frequencies of 0.7%, 10%, and 15% respectively. In the present study, Lophyohylini shows a complex chromosome evolution as well, which has led to complex karyotypic changes (*Osteopilus*, *Osteocephalus*, and *Phyllodytes*). The high rate of rearrangements observed in lophyohylines represents reliable evidence that fissions and reciprocal translocations would be one of the leading candidate mechanisms responsible for the increase of the 2n and FN found in *Osteopilus* and *Osteocephalus*.

Besides, Lophyohylini exhibits other interesting chromosomal variations not solely restricted to the number, size, and the activity of NORs or the occurrence of ITS, but related to the presence of chromosomal rearrangements and the intriguing fragile sites. The latter likely presents a still unreported diversity, since no clear pattern is evident, as they can vary in the content of heterochromatin or type. For instance, being DAPI^+^/CMA_3_^–^ in *Dryaderces pearsoni*, DAPI^–^/CMA_3_^+^ in *Nyctimantis siemersi*, neutral for both fluorochromes in *Trachycephalus typhonius*, or even can vary intrachromosomally as in *Osteopilus vastus*. The fluorochrome CMA_3_ is another promising character that has shown a centromeric CMA_3_^+^ pattern on almost all studied species of the tribe, excepting *Os*. *septentrionalis*, *Phyllodytes melanomystax*, *P*. *praeceptor*, and *T*. *dibernardoi*.

In a broad sense, regarding the reported cytogenetic variation, Lophyohylini resembles what is observed in direct-developing frogs of Brachycephaloidea and Hemiphractidae. Further studies in *Phytotriades* and *Tepuihyla* and an expanded sampling in the variable genus *Phyllodytes* would help to understand the puzzling cytogenetics of these intriguing frogs.

## Supporting information

S1 Fig DAPI and CMA_3_ staining in 16 species of Lophyohylini. (PDF)

S2 Fig Optimization of the haploid number (n) and the position of NORs (NORs) in Lophyohylini on the phylogenetic hypothesis of Blotto et al. [3]. (PDF)

S3 Fig Telomeric sequences in four species of Lophyohylini. A. *Phyllodytes gyrinaethes*. B. *Osteocephalus planiceps*. C. *O*. *taurinus*. D. *Nyctimantis arapapa*
(PDF)

S1 File Information of the specimens analyzed of each species. (PDF)

S1 Table Cytogenetic information in Lophyohylini. Differential techniques performed, chromosome number (2n) and NORs position observed in each species.
(PDF)

S2 Table Chromosome measurements of 21 species of Lophyohylini. Chromosome percentage relative to the haploid set (Chromosome Morphology) Centromeric Index ± Standard Deviation. m: metacentric; sm: submetacentric; st: subtelocentric; t: telocentric.
(PDF)

S3 Table Comparison of measures of pairs 5 and 6 between different species of Lophyohylini. (PDF)
