# Supplementary material for: Chromosome evolution in Lophyohylini (Amphibia, Anura, Hylinae)
Source: PLoS One. 2020 Jun 11;15(6):e0234331. doi: 10.1371/journal.pone.0234331 (PMC7289402; doi:10.1371/journal.pone.0234331)
Supplement: S1 Fig — (PDF) [file pone.0234331.s001.pdf]

S1 Fig. DAPI and CMA_3 staining in 16 species of Lophyohylini.

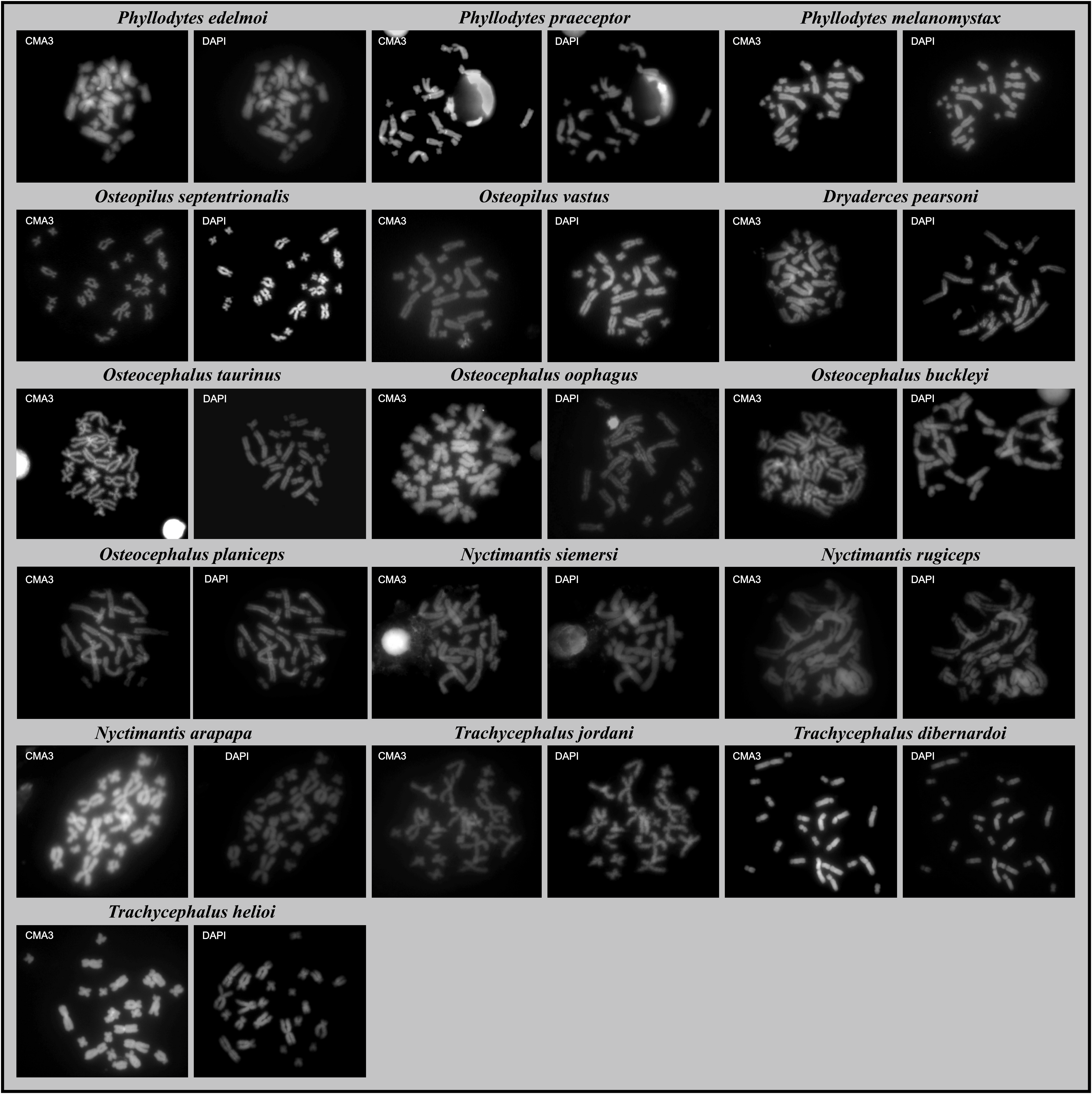
Phyllodytes edelmoi
CMA3
DAPI
Phyllodytes praeceptor
CMA3
DAPI
Phyllodytes melanomystax
CMA3
DAPI
Osteopilus septentrionalis
CMA3
DAPI
Osteopilus vastus
CMA3
DAPI
Dryaderces pearsoni
CMA3
DAPI
Osteocephalus taurinus
CMA3
DAPI
Osteocephalus oophagus
CMA3
DAPI
Osteocephalus buckleyi
CMA3
DAPI
Osteocephalus planiceps
CMA3
DAPI
Nyctimantis siemersi
CMA3
DAPI
Nyctimantis rugiceps
CMA3
DAPI
Nyctimantis arapapa
CMA3
DAPI
Trachycephalus jordani
CMA3
DAPI
Trachycephalus dibernardoi
CMA3
DAPI
Trachycephalus helioi
CMA3
DAPI
