# Supplementary material for: Chromosome evolution in Lophyohylini (Amphibia, Anura, Hylinae)
Source: PLoS One. 2020 Jun 11;15(6):e0234331. doi: 10.1371/journal.pone.0234331 (PMC7289402; doi:10.1371/journal.pone.0234331)
Supplement: S2 Fig — (PDF) [file pone.0234331.s002.pdf]

S2 Fig. Optimization of the haploid number (n) and the position of NORs (NORs) in Lophyohylini on the phylogenetic hypothesis of Blotto et al. (2020).

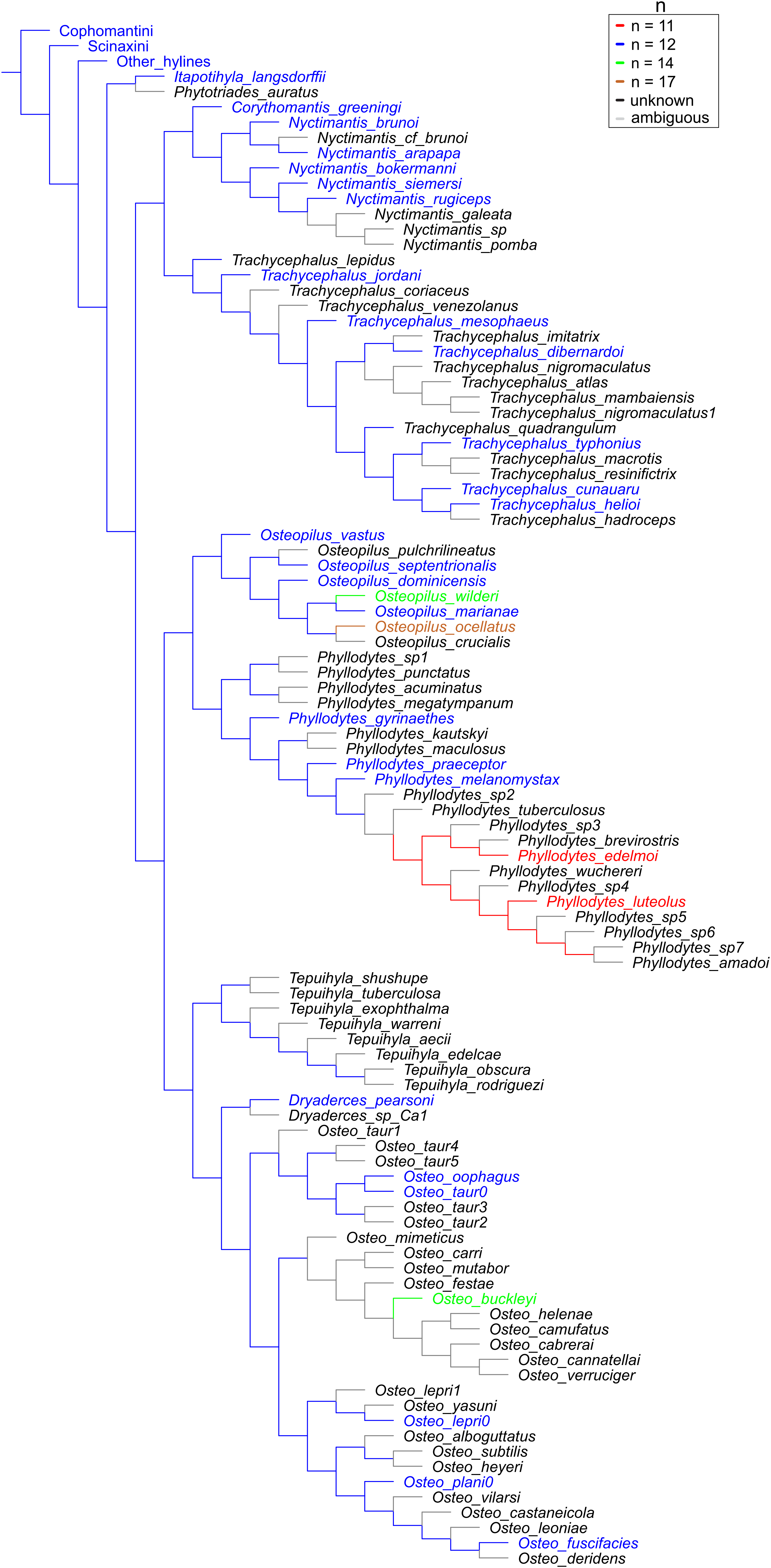

n
n = 11
n = 12
n = 14
n = 17
unknown
ambiguous
Cophomantini
Scinaxini
Other_hylines
Itapotihyla_langsdorffii
Phytotriades_auratus
Corythomantis_greeningi
Nyctimantis_brunoi
Nyctimantis_cf_brunoi
Nyctimantis_arapapa
Nyctimantis_bokermanni
Nyctimantis_siemersi
Nyctimantis_rugiceps
Nyctimantis_galeata
Nyctimantis_sp
Nyctimantis_pomba
Trachycephalus_lepidus
Trachycephalus_jordani
Trachycephalus_coriaceus
Trachycephalus_venezolanus
Trachycephalus_mesophaeus
Trachycephalus_imitatrix
Trachycephalus_dibernardoi
Trachycephalus_nigromaculatus
Trachycephalus_atlas
Trachycephalus_mambaiensis
Trachycephalus_nigromaculatus1
Trachycephalus_quadrangulum
Trachycephalus_typhonius
Trachycephalus_macrotis
Trachycephalus_resinifictrix
Trachycephalus_cunauaru
Trachycephalus_helioi
Trachycephalus_hadroceps
Osteopilus_vastus
Osteopilus_pulchrilineatus
Osteopilus_septentrionalis
Osteopilus_dominicensis
Osteopilus_wilderi
Osteopilus_marianae
Osteopilus_ocellatus
Osteopilus_crucialis
Phyllodytes_sp1
Phyllodytes_punctatus
Phyllodytes_acuminatus
Phyllodytes_megatympanum
Phyllodytes_gyrinaethes
Phyllodytes_kautskyi
Phyllodytes_maculosus
Phyllodytes_praeceptor
Phyllodytes_melanomystax
Phyllodytes_sp2
Phyllodytes_tuberculosus
Phyllodytes_sp3
Phyllodytes_brevirostris
Phyllodytes_edelmoi
Phyllodytes_wuchereri
Phyllodytes_sp4
Phyllodytes_luteolus
Phyllodytes_sp5
Phyllodytes_sp6
Phyllodytes_sp7
Phyllodytes_amadoi
Tepuihyla_shushupe
Tepuihyla_tuberculosa
Tepuihyla_exophthalma
Tepuihyla_warreni
Tepuihyla_aecii
Tepuihyla_edelcae
Tepuihyla_obscura
Tepuihyla_rodriguezi
Dryaderces_pearsoni
Dryaderces_sp_Ca1
Osteo_taur1
Osteo_taur4
Osteo_taur5
Osteo_oophagus
Osteo_taur0
Osteo_taur3
Osteo_taur2
Osteo_mimeticus
Osteo_carri
Osteo_mutabor
Osteo_festae
Osteo_buckleyi
Osteo_helenae
Osteo_camufatus
Osteo_cabrerai
Osteo_cannatellai
Osteo_verruciger
Osteo_lepri1
Osteo_yasuni
Osteo_lepri0
Osteo_alboguttatus
Osteo_subtilis
Osteo_heyeri
Osteo_plani0
Osteo_vilarsi
Osteo_castaneicola
Osteo_leoniae
Osteo_fuscifacies
Osteo_deridens

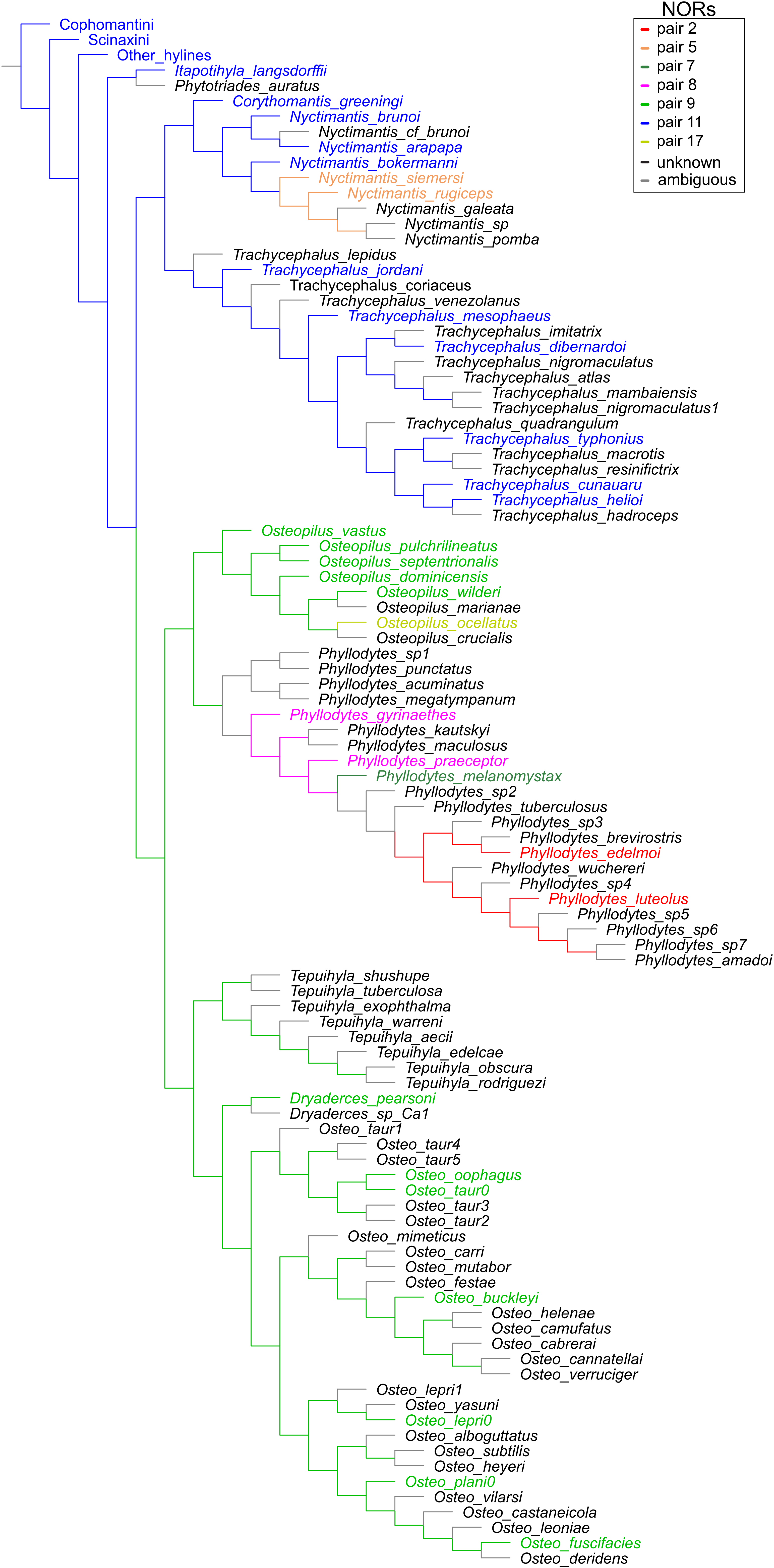
NORs
pair 2
pair 5
pair 7
pair 8
pair 9
pair 11
pair 17
unknown
ambiguous
Cophomantini
Scinaxini
Other_hylines
Itapotihyla_langsdorffii
Phytotriades_auratus
Corythomantis_greeningi
Nyctimantis_brunoi
Nyctimantis_cf_brunoi
Nyctimantis_arapapa
Nyctimantis_bokermanni
Nyctimantis_siemersi
Nyctimantis_rugiceps
Nyctimantis_galeata
Nyctimantis_sp
Nyctimantis_pomba
Trachycephalus_lepidus
Trachycephalus_jordani
Trachycephalus_coriaceus
Trachycephalus_venezolanus
Trachycephalus_mesophaeus
Trachycephalus_imitatrix
Trachycephalus_dibernardoi
Trachycephalus_nigromaculatus
Trachycephalus_atlas
Trachycephalus_mambaiensis
Trachycephalus_nigromaculatus1
Trachycephalus_quadrangulum
Trachycephalus_typhonius
Trachycephalus_macrotis
Trachycephalus_resinifictrix
Trachycephalus_cunauaru
Trachycephalus_helioi
Trachycephalus_hadroceps
Osteopilus_vastus
Osteopilus_pulchrilineatus
Osteopilus_septentrionalis
Osteopilus_dominicensis
Osteopilus_wilderi
Osteopilus_marianae
Osteopilus_ocellatus
Osteopilus_crucialis
Phyllodytes_sp1
Phyllodytes_punctatus
Phyllodytes_acuminatus
Phyllodytes_megatympanum
Phyllodytes_gyrinaethes
Phyllodytes_kautskyi
Phyllodytes_maculosus
Phyllodytes_praeceptor
Phyllodytes_melanomystax
Phyllodytes_sp2
Phyllodytes_tuberculosus
Phyllodytes_sp3
Phyllodytes_brevirostris
Phyllodytes_edelmoi
Phyllodytes_wuchereri
Phyllodytes_sp4
Phyllodytes_luteolus
Phyllodytes_sp5
Phyllodytes_sp6
Phyllodytes_sp7
Phyllodytes_amadoi
Tepuihyla_shushupe
Tepuihyla_tuberculosa
Tepuihyla_exophthalma
Tepuihyla_warreni
Tepuihyla_aecii
Tepuihyla_edelcae
Tepuihyla_obscura
Tepuihyla_rodriguezi
Dryaderces_pearsoni
Dryaderces_sp_Ca1
Osteo_taur1
Osteo_taur4
Osteo_taur5
Osteo_oophagus
Osteo_taur0
Osteo_taur3
Osteo_taur2
Osteo_mimeticus
Osteo_carri
Osteo_mutabor
Osteo_festae
Osteo_buckleyi
Osteo_helenae
Osteo_camufatus
Osteo_cabrerai
Osteo_cannatellai
Osteo_verruciger
Osteo_lepri1
Osteo_yasuni
Osteo_lepri0
Osteo_alboguttatus
Osteo_subtilis
Osteo_heyeri
Osteo_plani0
Osteo_vilarsi
Osteo_castaneicola
Osteo_leoniae
Osteo_fuscifacies
Osteo_deridens
