# Supplementary material for: Chromosome evolution in Lophyohylini (Amphibia, Anura, Hylinae)
Source: PLoS One. 2020 Jun 11;15(6):e0234331. doi: 10.1371/journal.pone.0234331 (PMC7289402; doi:10.1371/journal.pone.0234331)
Supplement: S3 Fig — A. Phyllodytes gyrinaethes. B. Osteocephalus planiceps. C. O. taurinus. D. Nyctimantis arapapa (PDF) [file pone.0234331.s003.pdf]

S3 Fig. Telomeric sequences in four species of Lophyohylini. A. *Phyllodytes gyrinaethes*. **B.** *Osteocephalus planiceps*. **C.** *O. taurinus*. **D.** *Nyctimantis arapapa*

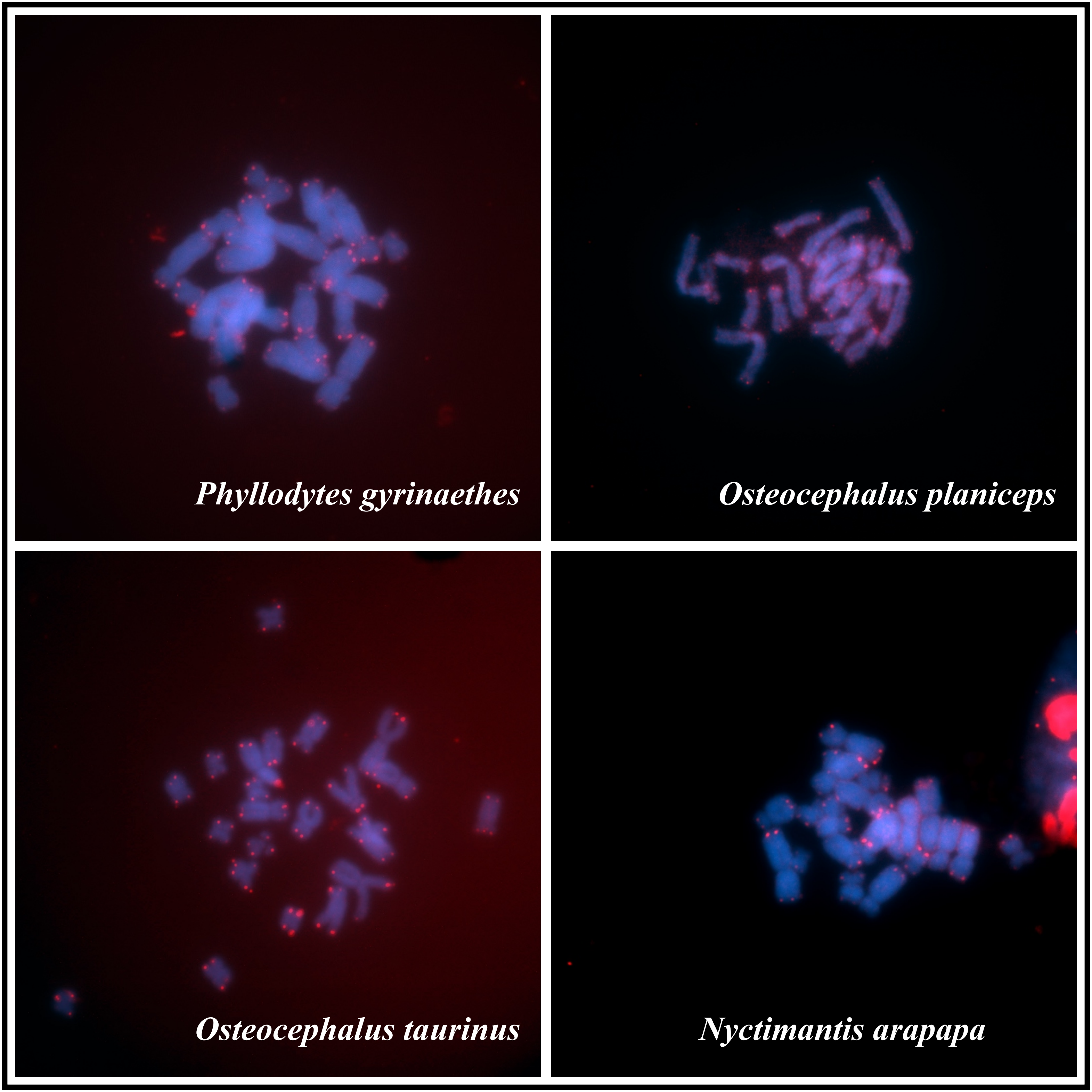
Phyllodytes gyrinaethes
Osteocephalus planiceps
Osteocephalus taurinus
Nyctimantis arapapa
