# Supplementary material for: Chromosome evolution in Lophyohylini (Amphibia, Anura, Hylinae)
Source: PLoS One. 2020 Jun 11;15(6):e0234331. doi: 10.1371/journal.pone.0234331 (PMC7289402; doi:10.1371/journal.pone.0234331)
Supplement: S1 File — (PDF) [file pone.0234331.s004.pdf]

S1 File. Information of the specimens analyzed of each species. Collected specimens are housed in the herpetological collections of Departamento de Zoologia, Instituto de Biociências, UNESP, Rio Claro, São Paulo, Brazil (CFBH); Laboratorio de Genética Evolutiva, Instituto de Biología Subtropical, Posadas, Misiones, Argentina (LGE); Museu Nacional de Rio de Janeiro, Rio de Janeiro, Brazil (MNRJ); Museu de Zoologia da Universidade Estadual de Santa Cruz, Ilhéus, Bahia, Brazil (MZUESC); Centro de Estudos Avançados da Biodiversidade, Universidade Federal do Pará, Belém, Pará, Brazil (PS), Museu Paraense Emílio Goeldi, Pará, Brazil (MPEG), Museo de Zoología de la Pontificia Universidad Católica del Ecuador (QCAZ), Dr. Euvaldo Marciano Junior field number (EMJ). Male (M), female (F), juvenile (J), undetermined (U). Geographic coordinates are in the system datum WGS84.

Dryaderces pearsoni.—BRAZIL: Pará: Juruti: Mutum camp. (02 36 48.3S; 56 11 40W), MPEG 22209 (F).

Itapotihyla langsdorffii.—ARGENTINA: Misiones: Iguazú: Puerto Iguazú (25 36 00S; 54 33 00W), LGE 20358 (M).

Nyctimantis arapapa.—BRAZIL: Bahia: Ilhéus, Acuípe, RPPN Nova Angélica (15 04 00S; 39 03 00W), EMJ 7 (F); Jequié (13 51 53.3S; 40 05 27.6W), PS 1053 (M).

Nyctimantis rugiceps.—ECUADOR: Pastaza: Lorocachi (01 37 09S; 75 58 56.6W), QCAZ 55662 (M).

Nyctimantis siemersi.—ARGENTINA: Corrientes: Ituzaingó: Reserva Santa María (27 31 53.8S; 56 35 41.6W), 11185 (F), 11192 (M), 11194 (M).

Osteocephalus buckleyi.—BRAZIL: Pará: Juruti: Barroso camp. (02 27 49.7S; 55 59 59.4W), PS 519 (F); Mutum camp. (02 36 48.3S; 56 11 40W), PS 463 (M), 465 (M).

Osteocephalus fuscifacies.—ECUADOR: Gualaquiza: Bomboiza (03 22 36.7S; 78 26 07.1W); QCAZA 74201 (M); 74202 (F).

Osteocephalus leprieurii.—BRAZIL: Pará: Juruti: Mutum camp. (02 36 48.3S; 56 11 40W), MPEG 37793 (M); Marabá, Salobo (05 50 16.8S; 50 30 57.6W), MPEG 37801 (M).

Osteocephalus oophagus.—BRAZIL: Pará: Juruti: Barroso camp. (02 27 49.7S; 55 59 59.4W), MPEG 22217 (U).

Osteocephalus planiceps.—ECUADOR: Morona Santiago: Vía Patuca-Puerto Morona, sendero cerca al Mirador de la Virgen (02 58 32.8S; 77 47 44.3W), QCAZA 53874 (J).

Osteocephalus taurinus.—BRAZIL: Pará state: Juruti: Barroso camp. (02 27 49.7S; 55 59 59.4W), PS 430 (M), 467 (M); Capiranga Plateau (02 30 20S; 56 11 06W), PS 452 (U).

Osteopilus septentrionalis.—Three Pet trade specimens (U).

Osteopilus vastus.—Four Pet trade specimens (U).

Phyllodytes edelmoi.—BRAZIL: Alagoas: Maceio: Serra da Saudinha (09 22 00S; 35 45 00W), PS 924 (M).

Phyllodytes gyrinaethes.—BRAZIL: Pernambuco: Lagoa dos Gatos, RPPN Pedra D'Anta (08 42 15.5S; 35 51 06.2W), CFBH 49633 (F), 49634 (M), 1971 (F).

Phyllodytes melanomystax.—BRAZIL: Bahia: Ilhéus: Ponta da Tulha (14 35 35S; 39 03 43W), MZUESC 14246 (M).

Phyllodytes praeceptor.—BRAZIL: Bahia: Una (15 11 02 S; 39 03 59W), EMJ one unnumbered specimen (U).

Trachycephalus dibernardoi.—ARGENTINA: Misiones: Guaraní: El Soberbio, Refugio Tangará (27 00 00S; 54 07 00W), LGE 19161 (F); Dept. 25 de Mayo: Puerto Londero, Arroyo Los Muertos y Ruta Provincial Nº 2 (27 22 11.5S; 54 24 30.4W), LGE 19162 (M).

Trachycephalus helioi.—BRAZIL: Pará: Juruti: Barroso camp. (02 27 49.7S; 55 59 59.4W), MNRJ 81611 (ex. MPEG 20506) (M); MPEG 20507 (M).

Trachycephalus jordani.—ECUADOR: Pichincha: Puerto Quito (00 07 33.6N; 79 15 10.8W), QCAZA 53914 (U).

Trachycephalus typhonius.—ARGENTINA: Misiones: Capital: Garupá, Barrio Santa Helena (27 27 53.5S; 55 51 52W), LGE 18974 (M); Posadas, Tiro Federal (27 21 36S; 55 54 24W), LGE 18963 (F); Villa Lanús, Refugio Don Lorenzo (27 25 29S; 55 52 08W), LGE 15133 (M); Iguazú: Puerto Iguazú (25 36 00S; 54 33 00W), LGE 18967 (M), LGE 15141 (M); Oberá: San Martín (27 27 50S; 55 20 17W), LGE 18979–80 (M); Santa Fe: Capital: Ruta provincial Nº 1, near to San José del Rincón (31 36 39.6S; 60 34 55.9W), LGE 18969 (F); San Javier: Itacaruaré (27 54 35S; 55 16 12W), LGE 18960 (U). BRAZIL: Pará: Belém, Cotijuba island (01 13 13.3S; 48 32 55.9W), PS 46 (M), 56 (M); Peixe Boi: Peixe Boi-3km norte (01 10 22S; 47 19 00W), PS 499 (F).
