# Supplementary material for: Chromosome evolution in Lophyohylini (Amphibia, Anura, Hylinae)
Source: PLoS One. 2020 Jun 11;15(6):e0234331. doi: 10.1371/journal.pone.0234331 (PMC7289402; doi:10.1371/journal.pone.0234331)
Supplement: S1 Table — Differential techniques performed, chromosome number (2n) and NORs position observed in each species. (PDF) [file pone.0234331.s005.pdf]

S1 Table. Cytogenetic information in Lophyohylini. Differential techniques performed, chromosome number (2n) and NORs position observed in each species.

Genus	Species	Reference	2n	Differential Techniques	NORs position
Corythomantis	***C. greeningi***	Kasahara et al., 2003	24	Ag-NORs, BrdU, C-bands, DAPI-CMA3, FISH(rDNA)	int 10p
		Schmid et al., 2018	24	C-bands	
Dryaderces	***D. piersoni***	present study	24	Ag-NORs, C-bands, DAPI/CMA_3, FISH(rDNA)	int 9q
Itapotihyla	***I. langsdorffii***	Kasahara et al., 2003, as *Osteocephalus langsdorffii*	24	Ag-NORs, C-bands, DAPI-CMA3, FISH(rDNA, tel)	int 10p
		Nunes and Fagundes, 2008	24	Ag-NORs	int 12q
		Gruber et al., 2012	24	Ag-NORs, C-bands, DAPI-CMA3, FISH(rDNA)	int 10p
		Schmid et al., 2018	24	C-bands	
		present study	24	Ag-NORs, C-bands, FISH(rDNA)	int 11p
Nyctimantis	***N. arapapa***	present study	24	Ag-NORs, C-bands, DAPI/CMA_3, FISH(rDNA, tel)	
	N. bokermanni	Gruber et al. 2012, as *Aparasphenodon bokermanni*	24	Ag-NORs, C-bands, DAPI-CMA_3, FISH(rDNA, tel)	ter 10q
		Schmid et al., 2018	24	C-bands	
	N. brunoi	Bogart, 1973, as *Corythomantis brunoi*	24		
		Kasahara et al., 1998, as *Aparasphenodon brunoi*	24	BrdU	
		Kasahara et al., 2003, as *Aparasphenodon brunoi*	24	Ag-NORs, BrdU, C-bands, DAPI-CMA3, FISH(rDNA)	ter 10q
		Nunes and Fagundes, 2008, *Aparasphenodon brunoi*	24		
		Schmid et al., 2018	24	C-bands, DAPI	
	N. rugiceps	present study	24	Ag-NORs, C-bands, DAPI/CMA_3, FISH(rDNA)	
	N. siemersi	Morand and Hernando, 1996, as *Argenteohyla siemersi pederseni*	24	Ag-NORs	per 4p, int 1q(oc), per 4p(oc)
		present study	24	Ag-NORs, DAPI/CMA3, C-bands, FISH(rDNA)	per 5p(oc)
Osteocephalus	***O. buckleyi***	present study	28	Ag-NORs, DAPI/CMA3, C-bands, FISH(rDNA, tel)	int 11q
	O. fuscifacies	present study	24	Ag-NORs	int 9q
	O. leprieurii	present study	24	C-bands	int 9q(sc)
	O. oophagus	present study	24	Ag-NORs, C-bands, DAPI/CMA_3, FISH(rDNA)	int 9q
	O. planiceps	present study	24	Ag-NORs, C-bands, DAPI/CMA_3, FISH(rDNA, tel)	int 9q
	O.* aff. *taurinus [a]	Anderson, 1996, as *O. taurinus*	24	Ag-NORs, C-bands	int 12p
	O.* aff. *taurinus [b]	Schmid et al., 2018, as *O. taurinus*	24	Q, DAPI/Mit	int 9q(DAPI/Mit)
	O. taurinus	present study	24	Ag-NORs, C-bands, DAPI/CMA_3, FISH(rDNA, tel)	int 9q
Osteopilus	***Os. ocellatus***	Cole, 1974			
		Anderson, 1996, as *Osteopilus brunneus*	34	Ag-NORs, C-bands, FISH(tel)	int 17[(1)]
		Schmid et al., 2018		Ag-NORs	ter 16 + subter 17
	Os. dominicensis	Anderson, 1996	24	Ag-NORs, C-bands	int 9p
		Schmid et al., 2018	24		
	Os. marianae	Anderson, 1996, as *Hyla marianae*	24		
		Schmid et al., 2018	24(ns)		
	Os. septentrionalis	Duellman and Cole, 1965	24		
		Cole, 1974	24		
		Schmid, 1978, as *Hyla septentrionalis*	24	Ag-NORs	prox 10q
		Anderson, 1996	24	Ag-NORs, C-bands, FISH(tel)	int 9p[(2)]
		Schmid and Steinlein, 2016	24	C-bands, 5-MeC	
		Schmid et al., 2018	24	Ag-NORs, C-bands, FISH(tel)	
		present study	24	Ag-NORs, C-bands, DAPI/CMA_3, FISH(rDNA)	int 9q
	Os. vastus	present study	24	Ag-NORs, C-bands, DAPI/CMA_3, FISH(rDNA)	int 9q
	Os. wilderi	Anderson, 1996, as *Hyla wilderi*	28	Ag-NORs, C-bands	int 9p
		Schmid et al., 2018	28		
Phyllodytes	***P. edelmoi***	Gruber et al. 2012	22	Ag-NORs, DAPI/CMA_3, FISH(tel)	ter 2q
		Schmid et al., 2018	22		
		present study	22	Ag-NORs, C-bands, DAPI/CMA_3, FISH(rDNA,tel)	ter 2q
	P. gyrinaethes	present study	24	Ag-NORs, DAPI/CMA_3,FISH(rDNA, tel)	ter 8q
	P. luteolus	Gruber et al 2012	22	Ag-NORs, DAPI/CMA_3, FISH(rDNA)	ter 2q
		Schmid et al., 2018	22		
	P. melanomystax	present study	24	Ag-NORs, FISH(tel)	ter 7q
	P. praeceptor	present study	24	Ag-NORs, DAPI/CMA_3	ter 8q
Trachycephalus	***T. cunuaru***	Gruber et al. 2012, as *Trachycephalus* sp.	24	Ag-NORs, C-bands, DAPI/CMA_3, FISH(rDNA, tel)	ter 10q
		Schmid et al., 2018	24	C-bands	
	T. dibernardoi	present study	24	Ag-NORs, C-bands, DAPI/CMA_3, FISH(rDNA)	ter 11q
	T. helioi	present study	24	Ag-NORs, C-bands, DAPI/CMA_3, FISH(rDNA)	ter 11q[(1)]
	T. jordani	present study	24	Ag-NORs, C-bands, DAPI/CMA_3, FISH(rDNA)	int 11q
	T. mesophaeus	Gruber et al 2012	24	Ag-NORs, C-bands, DAPI/CMA_3, FISH(rDNA, tel)	ter 10q
		Schmid et al., 2018	24	C-bands	

T. typhonius	Duellman and Cole, 1965, as *Phrynohyas spilomma*	24*		
	Rabello, 1970, as *Phrynohyas venulosa*	24		
	Bogart and Bogart, 1971, as *Phrynohyas venulosa*	24(ns)		
	Bogart, 1973, as *Phrynohyas venulosa*	24		
	Gruber et al. 2012	24	Ag-NORs, C-bands, DAPI/CMA_3, FISH(rDNA, tel)	ter 10q
	Schmid et al., 2018	24	C-bands	
	present study	24	Ag-NORs, C-bands, FISH(rDNA)	ter 11q(1)

***** inferred from the haploid number in meiosis
(ns) Image not shown in the original publication.
[a] specimens from Frenche Guaiana, probably corresponding to *O. taurinus* candidate species 5 of Jungfer et al., 2013
[b]specimens from Venezuela, unknown locality, probably corresponding to *O. taurinus* candidate species 2, 3 or 5 of Jungfer et al., 2013
(1) Additional polymorphic NORs
(2) NORs variation. In the intrachromosomal position on pair 9, and due to a translocation between one homologue of pair 6 and 9..
(oc) NORs on one chromosome
Q Quinacrine staining
DAPI/Mit DAPI/Mithramycin staining
(DAPI/Mit) Inferred by DAPI/Mit staining
5-Mec Inmunofluorescence anti body for detecting 5-Methylcytosine rich heterochromatin.
FISH Fluorescent in situ hybridization.
rDNA Ribosomal DNA probe.
tel Telomeric DNA probe.

References list

1. Anderson K. A karyological perspective on the monophyly of the hylid genus Osteopilus, In: R. Powell and R.W. Henderson (eds), Contributions to west Indian herpetology: A tribute to Albert Schwartz. Society for the Study of amphibians and reptiles. Ithaca, New York, USA. 1996; 12: 157–168.

2. Bogart JP, Bogart JE. Genetic compatibility experiments between some south American anuran amphibians. Herpetolog. 1971; 27:229–235.

3. Bogart JP. Evolution of anuran karyotypes. In: Vial JL (Ed.) Evolutionary Biology of the Anurans. University of Missouri Press, USA. 1973; Pp. 337–349.

4. Cole CJ. Chromosome evolution in selected treefrogs, including Casque-Head species (Pternohyla, Triprion, Hyla and Smilisca). American Museum Novitates. 1974; 2541: 1–10.

5. Duellman WE, Cole CJ. Studies of Chromosomes of Some Anuran Amphibians (Hylidae and Centrolenidae). Systematic Zoology. 1965; 14(2) 139–143.

6. Gruber SL, Haddad CFB, Kasahara S, 2012. Karyotype analysis of seven species of the tribe Lophiohylini (Hylinae, Hylidae, Anura), with conventional and molecular cytogenetic techniques. Comparative Cytogenetics, 6:409–423.

7. Kasahara S, Silva APZ, Gruber SL. Use of lymphocyte cultures for BrdU replication banding patterns in anuran species (Amphibia). Genet Mol Biol. 1998; 21:471–476

8. Kasahara S, Zampieri Silva AP, Gruber SL, Haddad CFB. Comparative cytogenetic analysis on four tree frog species (Anura, Hylidae, Hylinae) from Brazil. Cytogenet Genome Res. 2003

9. Morand M, Hernando A. Cariotipo y región organizadora del nucleolo en Argenteohyla siemersi pederseni (Anura: Hylidae). Facena. 1996; 12: 141–143.

10. Nunes RDRA, Fagundes V. Cariótipos de oito espécies de anfíbios das subfamílias Hylinae e Phyllomedusinae (Anura, Hylidae) do Espírito Santo, Brasil. Bol Do Mus Biol Mello Leitão Nov Série. 2008; 23: 21–36.

11. Rabello MN. Chromosomal studies in Brazilian anurans. Caryologia. 1970; 23(1): 45–59.

12. Schmid M, Steinlein C (b). Chromosome Banding in Amphibia. XXXIII. Demonstration of 5-Methylcytosine-Rich Heterochromatin in Anura. Cytogenet Genome Res. 2016; 148(1): 35–43.

13. Schmid M, Steinlein C, Haaf T, Feightinger W, Guttenbach M, Bogart JP, et al. The Arboranan frogs: evolution, biology, and cytogenetics. Cytogenet Genome Res. 2018; 155:1–325.

14. Schmid M. Chromosome banding in Amphibia I. Constitutive heterochromatin and nucleolus organizer regions in Bufo and Hyla. Chromosoma (Berl.). 1978; 66:361–88.
