# Supplementary material for: Chromosome evolution in Lophyohylini (Amphibia, Anura, Hylinae)
Source: PLoS One. 2020 Jun 11;15(6):e0234331. doi: 10.1371/journal.pone.0234331 (PMC7289402; doi:10.1371/journal.pone.0234331)
Supplement: S2 Table — Chromosome percentage relative to the haploid set (Chromosome Morphology) Centromeric Index ± Standard Deviation. m: metacentric; sm: submetacentric; st: subtelocentric; t: telocentric. (PDF) [file pone.0234331.s006.pdf]

**S2 Table. Chromosome measurements of 21 species of Lophyohylini.** Chromosome percentage relative to the haploid set (Chromosome Mophology) Centromeric Index ± Standard Deviation. m: metacentric; sm: submetacentric; st: subtelocentric; t: telocentric.

| ***Species of Lophiohylini*** | ***Chromosome Pair*** | | | | | | | | | | | | | |
|---|---|---|---|---|---|---|---|---|---|---|---|---|---|---|
| | ***1*** | ***2*** | ***3*** | ***4*** | ***5*** | ***6*** | ***7*** | ***8*** | ***9*** | ***10*** | ***11*** | ***12*** | ***13*** | ***14*** |
| ***Phyllodytes edelmoi*** | 16.65 **(m)** .46 ± .02 | 13.91 **(sm)** .36 ± .03 | 11.09 **(sm)** .35 ± .03 | 10.96 **(m)** .43 ± .03 | 9.89 **(sm)** .35 ± .02 | 8.45 **(sm)** .36 ± .02 | 7.92 **(sm)** .38 ± .03 | 6.67 **(sm)** .35 ± .02 | 5.19 **(m)** .45 ± .04 | 4.85 **(m)** .45 ± .02 | 4.42 **(m)** .45 ± .03 | | | |
| ***Phyllodytes gyrinaethes*** | 17.40 **(m)** .47 ± .01 | 12.90 **(m)** .40 ± .01 | 10.90 **(sm)** .28 ± .02 | 10.80 **(sm)** .32 ± .02 | 10.20 **(sm)** .26 ± .02 | 7.20 **(st)** .24 ± .03 | 6.80 **(m)** .39 ± .02 | 5.80 **(sm)** .36 ± .03 | 5.20 **(m)** .39 ± .04 | 4.80 **(m)** .43 ± .02 | 4.20 **(m)** .40 ± .04 | 3.70 **(m)** .41 ± .05 | | |
| ***Phyllodytes praeceptor*** | 14.20 **(m)** .40 ± .03 | 12.40 **(sm)** .35 ± .01 | 11.60 **(st)** .18 ± .01 | 10.80 **(sm)** .32 ± .03 | 9.87 **(st)** .18 ± .03 | 9.37 **(st)** .24 ± .03 | 8.50 **(st)** .17 ± .01 | 7.13 **(sm)** .28 ± .01 | 5.32 **(sm)** .37 ± .01 | 4.08 **(m)** .40 ± .03 | 4.00 **(sm)** .37 ± .02 | 2.76 **(m)** .48 ± .02 | | |
| ***Phyllodytes melanomystax*** | 18.20 **(m)** .42 ± .01 | 14.10 **(sm)** .36 ± .02 | 12.30 **(sm)** .34 ± .01 | 10.30 **(st)** .18 ± .01 | 10.10 **(sm)** .30 ± .03 | 8.60 **(m)** .42 ± .01 | 7.40 **(sm)** .27 ± .01 | 5.30 **(m)** .41 ± .01 | 4.30 **(m)** .40 ± .01 | 3.70 **(m)** .45 ± .02 | 3.10 **(m)** .44 ± .05 | 2.60 **(m)** .47 ± .02 | | |
| ***Itapotihyla langsdorffii*** | 15.40 **(m)** .45 ± .01 | 12.60 **(m)** .39 ± .01 | 10.80 **(sm)** .33 ± .02 | 10.80 **(st)** .25 ± .01 | 9.80 **(sm)** .31 ± .02 | 7.50 **(st)** .19 ± .01 | 6.40 **(sm)** .36 ± .02 | 6.40 **(m)** .39 ± .02 | 6.00 **(m)** .38 ± .01 | 5.50 **(m)** .40 ± .01 | 4.60 **(m)** .43 ± .02 | 4.00 **(m)** .41 ± .03 | | |
| ***Osteopilus septentrionalis*** | 16.60 **(m)** .45 ± .02 | 13.40 **(sm)** .36 ± .03 | 11.10 **(sm)** .32 ± .01 | 12.00 **(st)** .22 ± .01 | 9.41 **(sm)** .29 ± .02 | 8.00 **(st)** .18 ± .01 | 6.30 **(sm)** .32 ± .03 | 5.70 **(m)** .42 ± .06 | 5.30 **(m)** .45 ± .01 | 4.70 **(m)** .43 ± .03 | 4.20 **(m)** .43 ± .03 | 3.30 **(m)** .43 ± .06 | | |
| ***Osteopilus vastus*** | 16.20 **(m)** .43 ± .03 | 13.50 **(m)** .38 ± .02 | 11.10 **(sm)** .32 ± .02 | 11.50 **(st)** .20 ± .03 | 9.80 **(sm)** .31 ± .03 | 8.00 **(st)** .16 ± .02 | 6.10 **(sm)** .34 ± .03 | 5.30 **(m)** .41 ± .03 | 5.10 **(m)** .46 ± .03 | 4.80 **(m)** .46 ± .03 | 4.10 **(m)** .43 ± .02 | 3.80 **(m)** .47 ± .04 | | |
| ***Dryaderces pearsoni*** | 16.03 **(m)** .46 ± .02 | 12.91 **(m)** .41 ± .01 | 11.47 **(sm)** .32 ± .01 | 9.67 **(st)** .22 ± .01 | 9.14 **(sm)** .34 ± .03 | 8.13 **(sm)** .27 ± .02 | 6.99 **(m)** .47 ± .02 | 6.34 **(m)** .49 ± .01 | 5.60 **(sm)** .35 ± .02 | 5.09 **(m)** .46 ± .03 | 4.40 **(m)** .46 ± .02 | 4.23 **(sm)** .37 ± .03 | | |
| ***Osteocephalus buckleyi*** | 11.30 **(st)** .24 ± .02 | 10.70 **(sm)** .30 ± .03 | 8.50 **(st)** .22 ± .03 | 7.50 **(t)** .00 ± .00 | 7.80 **(st)** .21 ± .03 | 7.20 **(t)** .00 ± .00 | 6.80 **(t)** .00 ± .00 | 6.50 **(m)** .45 ± .03 | 6.60 **(sm)** .33 ± .04 | 6.20 **(m)** .38 ± .03 | 5.80 **(sm)** .37 ± .07 | 5.70 **(m)** .45 ± .02 | 5.40 **(m)** .46 ± .03 | 3.80 **(m)** .47 ± .02 |
| ***Osteocephalus fuscifacies*** | 15.65 **(m)** .47 ± .02 | 11.94 **(m)** .43 ± .04 | 10.84 **(sm)** .33 ± .04 | 10.61 **(st)** .24 ± .02 | 9.47 **(sm)** .35 ± .01 | 7.74 **(sm)** .26 ± .02 | 6.11 **(sm)** .37 ± .03 | 6.12 **(m)** .44 ± .05 | 6.33 **(m)** .47 ± .04 | 5.63 **(m)** .44 ± .01 | 5.24 **(m)** .47 ± .02 | 4.33 **(m)** .45 ± .03 | | |
| ***Osteocephalus leprieurii*** | 15.90 **(m)** .47 ± .01 | 12.60 **(m)** .41 ± .01 | 10.60 **(sm)** .32 ± .02 | 10.80 **(st)** .20 ± .04 | 9.43 **(sm)** .32 ± .00 | 8.20 **(sm)** .28 ± .02 | 6.40 **(m)** .38 ± .04 | 6.30 **(m)** .49 ± .01 | 6.30 **(m)** .44 ± .02 | 4.80 **(m)** .46 ± .01 | 4.80 **(m)** .45 ± .06 | 4.10 **(m)** .43 ± .01 | | |
| ***Osteocephalus oophagus*** | 14.30 **(m)** .47 ± .02 | 13.30 **(m)** .42 ± .02 | 11.80 **(sm)** .34 ± .02 | 10.30 **(st)** .25 ± .01 | 9.00 **(sm)** .32 ± .01 | 8.10 **(sm)** .29 ± .01 | 6.50 **(m)** .38 ± .02 | 6.10 **(m)** .43 ± .04 | 5.60 **(m)** .45 ± .05 | 5.60 **(m)** .47 ± .03 | 5.40 **(m)** .40 ± .04 | 4.10 **(m)** .47 ± .03 | | |
| ***Osteocephalus planiceps*** | 15.70 **(m)** .47 ± .01 | 12.40 **(sm)** .34 ± .00 | 10.40 **(sm)** .28 ± .05 | 11.70 **(st)** .23 ± .03 | 9.81 **(sm)** .30 ± .05 | 7.80 **(st)** .23 ± .00 | 6.70 **(sm)** .31 ± .01 | 5.70 **(m)** .41 ± .01 | 5.60 **(m)** .45 ± .01 | 5.10 **(m)** .39 ± .03 | 5.10 **(m)** .41 ± .01 | 3.90 **(m)** .48 ± .00 | | |
| ***Osteocephalus taurinus*** | 15.30 **(m)** .47 ± .01 | 12.00 **(m)** .40 ± .02 | 10.90 **(sm)** .34 ± .02 | 10.70 **(st)** .25 ± .02 | 9.00 **(sm)** .34 ± .02 | 7.30 **(st)** .24 ± .02 | 6.70 **(m)** .40 ± .03 | 6.20 **(m)** .45 ± .02 | 6.10 **(m)** .44 ± .03 | 6.00 **(m)** .41 ± .04 | 5.60 **(m)** .44 ± .04 | 4.10 **(m)** .47 ± .02 | | |
| ***Nyctimantis siemersi*** | 17.60 **(m)** .45 ± .02 | 14.70 **(m)** .41 ± .03 | 11.70 **(sm)** .33 ± .01 | 9.82 **(st)** .23 ± .01 | 10.60 **(sm)** .30 ± .02 | 7.41 **(st)** .21 ± .02 | 6.50 **(sm)** .29 ± .02 | 6.00 **(m)** .43 ± .01 | 4.80 **(sm)** .34 ± .02 | 4.40 **(m)** .43 ± .02 | 3.30 **(sm)** .35 ± .02 | 3.10 **(sm)** .36 ± .02 | | |
| ***Nyctimantis rugiceps*** | 16.54 **(m)** .46 ± .03 | 14.93 **(m)** .40 ± .00 | 11.39 **(sm)** .30 ± .03 | 11.30 **(st)** .24 ± .01 | 10.44 **(sm)** .31 ± .03 | 7.82 **(st)** .19 ± .03 | 5.64 **(m)** .44 ± .02 | 5.34 **(sm)** .31 ± .01 | 4.99 **(sm)** .32 ± .03 | 4.47 **(sm)** .37 ± .05 | 3.97 **(sm)** .34 ± .02 | 3.10 **(m)** .40 ± .00 | | |
| ***Nyctimantis arapapa*** | 14.90 **(m)** .46 ± .02 | 12.50 **(sm)** .33 ± .05 | 11.40 **(m)** .40 ± .02 | 11.10 **(sm)** .28 ± .02 | 10.00 **(sm)** .32 ± .03 | 8.30 **(sm)** .28 ± .02 | 6.50 **(sm)** .37 ± .01 | 6.40 **(m)** .39 ± .03 | 5.50 **(sm)** .37 ± .05 | 5.30 **(sm)** .31 ± .04 | 4.50 **(m)** .38 ± .01 | 3.80 **(m)** .38 ± .04 | | |
| ***Trachycephalus jordani*** | 16.20 **(m)** .44 ± .02 | 13.90 **(m)** .39 ± .03 | 11.40 **(sm)** .29 ± .08 | 10.50 **(sm)** .26 ± .04 | 9.20 **(sm)** .32 ± .01 | 7.60 **(st)** .20 ± .02 | 6.40 **(sm)** .37 ± .03 | 5.70 **(m)** .38 ± .05 | 5.60 **(sm)** .36 ± .02 | 5.50 **(sm)** .37 ± .05 | 4.10 **(m)** .47 ± .06 | 3.60 **(m)** .44 ± .03 | | |
| ***Trachycephalus dibernardoi*** | 14.90 **(m)** .45 ± .02 | 11.60 **(m)** .40 ± .01 | 11.30 **(sm)** .37 ± .01 | 10.90 **(st)** .24 ± .01 | 10.70 **(sm)** .31 ± .01 | 8.00 **(st)** .19 ± .01 | 7.10 **(sm)** .30 ± .02 | 5.90 **(m)** .43 ± .01 | 5.80 **(sm)** .36 ± .01 | 5.10 **(m)** .41 ± .01 | 4.40 **(sm)** .33 ± .02 | 4.30 **(sm)** .36 ± .02 | | |
| ***Trachycephalus helioi*** | 15.00 **(m)** .45 ± .02 | 12.90 **(m)** .45 ± .01 | 11.20 **(sm)** .35 ± .02 | 11.10 **(st)** .25 ± .02 | 9.90 **(sm)** .29 ± .02 | 7.30 **(st)** .21 ± .01 | 6.90 **(sm)** .35 ± .02 | 5.90 **(m)** .47 ± .03 | 5.50 **(m)** .38 ± .03 | 5.30 **(m)** .43 ± .03 | 4.70 **(sm)** .37 ± .05 | 4.20 **(m)** .46 ± .02 | | |
| ***Trachycephalus typhonius*** | 15.10 **(m)** .45 ± .01 | 12.30 **(m)** .40 ± .01 | 10.70 **(sm)** .35 ± .01 | 10.30 **(st)** .24 ± .02 | 9.40 **(sm)** .32 ± .02 | 8.10 **(st)** .20 ± .01 | 6.80 **(sm)** .34 ± .01 | 6.60 **(m)** .42 ± .02 | 5.70 **(sm)** .35 ± .02 | 5.70 **(m)** .42 ± .02 | 5.30 **(sm)** .34 ± .02 | 4.00 **(m)** .42 ± .02 | | |
