# Supplementary material for: Chromosome evolution in Lophyohylini (Amphibia, Anura, Hylinae)
Source: PLoS One. 2020 Jun 11;15(6):e0234331. doi: 10.1371/journal.pone.0234331 (PMC7289402; doi:10.1371/journal.pone.0234331)
Supplement: S3 Table — (PDF) [file pone.0234331.s007.pdf]

S3 Table. Comparison of measures of pairs 5 and 6 between different species of Lophyohylini

Species	%Set			Reference
	pair 5	pair 6	dif	
Corythomantis greeningi	9,53	7,63	1,9	Kasahara et al., 2003
Dryaderces pearsoni	9,14	8,13	1,01	Suarez et al., 2019
Itapotihyla langsdorffii	10,91	7,58	3,33	Kasahara et al., 2003
	9,82	7,68	2,14	Gruber et al 2012
	9,8	7,5	2,3	Suarez et al., 2019
Nyctimantis arapapa	10	8,3	1,7	Suarez et al., 2019
Nyctimantis bokermanni	9,48	7,48	2	Gruber et al 2012
Nyctimantis brunoi	10,74	7,5	3,24	Kasahara et al., 2003
Nyctimantis rugiceps	10,44	7,82	2,62	Suarez et al., 2019
Nyctimantis siemersi	10,50	9,59	0,91	Morand y Hernando, 1996*
	10,6	7,41	3,19	Suarez et al., 2019
Osteocephalus leprieurii	9,4	8,2	1,2	Suarez et al., 2019
Osteocephalus oophagus	9	8,1	0,9	Suarez et al., 2019
Osteocephalus planiceps	9,8	7,8	2	Suarez et al., 2019
Osteocephalus* aff. *taurinus	9,32	8,17	1,16	Anderson, 1996*
Osteocephalus taurinus	9	7,3	1,7	Suarez et al., 2019
Osteopilus dominicensis	8,44	8,38	0,06	Anderson, 1996*
Osteopilus marianae	10,83	7,37	3,46	Anderson, 1996*
Osteopilus septentrionalis	9,49	8,12	1,37	Anderson, 1996*
	9,4	8	1,4	Suarez et al., 2019
Osteopilus vastus	9,8	8	1,8	Suarez et al., 2019
Phyllodytes gyrinaethes	10,2	7,2	3	Suarez et al., 2019
Phyllodytes melanomystax	10,1	8,6	1,5	Suarez et al., 2019
***Phyllodytes* sp.**	9,87	9,37	0,5	Suarez et al., 2019
Trachycephalus dibernardoi	10,7	8	2,7	Suarez et al., 2019
Trachycephalus helioi	9,9	7,3	2,6	Suarez et al., 2019
Trachycephalus jordani	9,2	7,6	1,6	Suarez et al., 2019
Trachycephalus mesophaeus	9,04	7,98	1,06	Gruber et al 2012
Trachycephalus cunauaru	9,18	7,81	1,37	Gruber et al 2012
Trachycephalus typhonius	10,06	8,16	1,9	Gruber et al 2012
	9,4	8,1	1,3	Suarez et al., 2019
Trachycephalus venulusos	9,28	7,89	1,39	Bogart, 1973*

max-min: 3.46–0.06; SD: 0.87

*measements obtained from the original publication
